# Glucose Tolerance Test (GTT) Curves of Water and Ethanol Extracts of Whole Body Apis mellifera jemenitica

**DOI:** 10.12688/f1000research.166622.2

**Published:** 2026-01-10

**Authors:** Raaih Marwae Ahmad Asseri, Mohamed Adam Ali Ismail, Hamed A Ghramh, Wed Mohammed Ali Alarjani, Tarik El-Sayed Ali Ismail, Mogbel A A El-Niweiri, Mohammed Mohammed

**Affiliations:** 1 Department of Chemical Engineering, King Khalid University, Abha, Aseer Province, Saudi Arabia; 2 Department of Biology, King Khalid University, Abha, Aseer Province, Saudi Arabia; 3 Honeybees and their Products Research Center, King Khalid University, Abha, Aseer Province, Saudi Arabia; 4 Department of Chemistry- Preparatory Year Program, Batterjee Medical College, Abha, Aseer Region, Saudi Arabia; 5 Department of Chemistry, King Khalid University, Abha, Aseer Province, Saudi Arabia

**Keywords:** Chemical composition of honeybees, biological activity, IR spectrometry, spectral database of organic compounds., Biochemistry and chemical ecology, Hive product science

## Abstract

**Background:**

This article explored the chemical constituents of water and ethanol extracts from the whole body of *A. m. jemenitica* honeybee drones and workers, investigating their effects on glucose tolerance test (GTT) curves through feeding experiments conducted on *Oryctolagus cuniculus* male rabbits.

**Methods:**

Chemical analysis of the extracts was performed using infrared (IR) spectroscopy alongside the spectral database of organic compounds.

**Results:**

The water extract revealed a richer diversity of natural products (38 compounds) compared to the ethanol extract (12 compounds). Notably, the water extract comprised various bioactive molecules, including sugars and their derivatives, phenolic compounds, alkaloids, quinones, amino acid derivatives, dipeptides, and organometallic compounds. In contrast, the ethanol extract primarily contained sugar derivatives, phenolic compounds, alkaloids, and pesticides. The water extract decreased the blood glucose level and transformed the GTT curve from a convex to a concave shape.

**Conclusion:**

The blood glucose-lowering effect of the water extract may be attributed to the anti-diabetic properties of its dipeptides, phenolic compounds and alkaloid contents.

## 1. Introduction

Entomotherapy refers to the use of insects and their products for the treatment of various diseases. Insects are known to possess bodies and products that are rich in bioactive compounds, enabling them to act as antimicrobial, antioxidant, anticancer, and immunomodulatory agents ( Guarnieri et al., 2022; Khayrova et al., 2022; Seabrooks and Hu, 2017). This practice is prevalent in several cultures, notably in countries such as China, India, Thailand, and parts of Africa. Traditionally, insects have been used to treat a variety of ailments, including kidney diseases, digestive tract disorders, asthma, chronic cough, liver issues, rheumatoid conditions, and tooth pain ( Siddiqui et al., 2023).

Honeybee larvae and pupae have been reported to be effective in treating skin wounds, gastrointestinal issues, and mental health distress ( Meda et al., 2004). According to Choi (2021), extracts from the pupae of A. me. ligustica drones demonstrated a range of biological activities, including antimicrobial, antioxidant, anti-inflammatory, anti-diabetic, anti-obesity benefits, skin whitening effects, prevention of hair loss, and an increase in blood testosterone levels ( Choi, 2021). Both the brood (larvae and pupae) and adult honeybees are known to contain various bioactive and nutritious compounds, such as proteins, essential amino acids, saturated and monounsaturated fatty acids, vitamins, minerals, and antioxidants. The nutritional composition of Apis mellifera larvae and pupae is influenced by factors including their diet, health, age, species, climatic conditions, and seasonal variations ( Guiné et al., 2022; Ghosh et al., 2016). However, it’s also important to note that the whole body of Apis mellifera can contain environmental contaminants like pesticides, heavy metals, and veterinary drugs, which could impact their safety and efficacy ( Traynor et al., 2021; Savarino et al., 2020; Borkovcová et al., 2022). In a 2024 study, Abedelmaksoud and colleagues investigated the nutritional composition of drone and worker honeybees at both the pupal and adult stages, quantifying levels of proteins, carbohydrates, amino acids, and minerals ( Abedelmaksoud et al., 2024).

The Glucose Tolerance Test (GTT) is a diagnostic procedure designed to assess a patient’s ability to metabolize glucose effectively. It plays a crucial role in identifying conditions such as diabetes mellitus, insulin resistance, acromegaly (reactive hypoglycemia), and disturbances in carbohydrate metabolism, as well as evaluating pancreatic beta-cell function ( Eyth et al., 2024).

The Oral Glucose Tolerance Test (OGTT) consists of several key steps: 1) **Fasting Preparation:** The study subject must arrive at the laboratory in a fasting state, ideally in the morning; 2) **Initial Blood Sample:** A baseline blood sample is collected to measure the fasting blood glucose level; 3) **Glucose Administration:** The study subject is given an oral glucose load, typically 70 grams dissolved in 250 mL of water or 1.75 grams per kilogram of body weight for children; and 4) **Subsequent Blood Sampling:** Blood samples are taken every 30 minutes for a duration of two to three hours to monitor changes in blood glucose levels. It is essential to collect blood samples in fluoride-containing tubes to prevent a decrease in glucose concentration. The fluoride acts as an inhibitor of glycolysis by blocking enolase, thereby preserving the glucose levels for accurate measurement ( Eyth et al., 2024).

This article aimed to: 1) investigate the chemical constituents of water and ethanol extracts of adult *Apis mellifera jemenitica* honeybees to assess their potential medicinal values; 2) explore the effects of these extracts on the glucose tolerance test (GTT) curves and blood glucose levels using *Oryctolagus cuniculus* rabbits as experimental subjects; and 3) identify which specific chemical constituents of the extracts may account for their effects on the GTT curve. To our knowledge, this study is unique as it is the first to examine the bioactive compounds, nutritive biomolecules, and environmental contaminants in the whole bodies of *A. m. jemenitica* workers and drones beside its bioactivity as regulator of blood glucose level.

## 2. Material and methods

### 2.1 Ethical clearance and informed consent

This research was carried out after being approved by the research ethics committee of King Khalid University under the license number (ECM#2024-2004). The issued ethical license is entitled as Effectiveness of Extracts of sugar ants and Apis mellifera jemenitica in the treatment of diabetes mellitus. The *Oryctolagus cuniculus* rabbits were used as animal models for the treatment of diabetes mellitus. However, the Oryctolagus cuniculus rabbits are not mentioned in title of the ethical license, but it was stated in the proposal sent to the ethical committee. Regarding the informed consent, we are the owners of the honeybees and the rabbits. The honeybees were obtained from our apiaries and the rabbits were bought from the local market private veterinary clinics.

### 2.2 Research design and the study groups

This study followed a pilot true experimental research design. It included a control group (four rabbits) and two experimental groups (two rabbits each). Initially, all four rabbits served as the control group before being divided into two experimental groups of two rabbits each. The four rabbits were from the species of *Oryctolagus cuniculus* rabbits as stated by the owner and they were confirmed using the Google Lens at: https://lens.google/#shopping.

### 2.3 Preparation of the Honeybee samples and the extraction in water and ethanol

The study sample consisted of adult workers and drones of *A. m. jemenitica* with the age of, approximately, 30 days. The workers and drones were collected from a single hive at morning in the summer season and when the bees were actively foraging. To collect bee samples from a hive, specific steps were followed: 1) Smoke was utilized to calm the bees and reduce aggression; 2) The hive was opened gently, ensuring that the queen was absent; 3) A frame was removed from the hive and examined to confirm the absence of the queen; 4) The frame was placed next to a sample collection box; and 5) The bees were gently shaken into the box and quickly closed to prevent them from flying away. The gentle shaking aimed to minimize the level of monoamine neurotransmitters (dopamine, octopamine, and serotonin) in the hemolymph to prepare the honeybees for euthanasia. Euthanasia of the studied honeybees was achieved by transferring them into liquid nitrogen, ensuring rapid death due to the extremely low temperature, similar to using dry ice for killing honeybees ( Mutinelli, 2021).

The deceased honeybees were divided into two parts (10 g each) and soaked in 50 mL of water or ethanol. The soaked honeybee samples were then incubated for 48 hours at 35°C while shaking to facilitate effective extraction and they were immediately analyzed after the incubation period ( Mutinelli, 2021).

### 2.4 The experimental Oryctolagus cuniculus rabbits and the blood sampling

The four *Oryctolagus cuniculus* rabbits were housed in pairs (two rabbits per cage) for a one-week acclimatization period. Following adaptation, all rabbits received an oral glucose (MERCK; G8270-1KG) load to establish baseline glucose tolerance test (GTT) curves, which served as control data. After a one-week washout period, the rabbits were divided into two experimental groups (n=2 per group) to assess the effects of aqueous and ethanolic honeybee extracts on GTT profiles.

*2.4.1 Inclusion and exclusion criteria*

Only physically healthy, 3 month age and male *Oryctolagus cuniculus* rabbits were included in this study.

*2.4.2 Blinding*

Asseri RM and Mohammed MEA were responsible for looking after the rabits and blood sampling. All the rabbits were put in the same conditions in one animal house at room temperature.

### 2.5 Blood sampling and measurement of blood glucose

Blood samples were collected from the central ear artery after applying a local anesthetic and disinfecting the area with 70% ethanol. A few drops of blood were placed directly onto a Contour TS glucometer (Bayer HealthCare, Germany) and the glucose concentration (mg/100 mL) was recorded.

### 2.6 GTT procedure

Four key steps were implemented to conduct the Glucose Tolerance Test (GTT): 1) **Overnight Fasting:** Rabbits were subjected to fasting overnight to establish a baseline; 2) **Fasting Blood Sample:** A fasting blood glucose sample was collected following the fasting period; 3) **Glucose Administration:** Each rabbit was given a glucose load of 1 g/kg orally alone or with 1 g/kg of each honeybee extract; and 4) **Post-Load Sampling:** After the glucose administration, blood samples were taken every 30 minutes for a duration of three hours to monitor glucose levels ( Eyth et al., 2024; Moro and Magnan, 2025).

### 2.7 IR spectrometry

The IR scanning was conducted using the Agilent Cary 630 FTIR Spectrometer (Agilent, USA), accompanied by the Agilent MicroLab software suite. A single drop of each extract was placed on the sampler, and the scanning was performed across a spectral range of 7000 to 350 cm ^−1^. The resulting spectra highlighted specific peak values, which were subsequently recorded for analysis according the procedure of the manufacturer ( Agilent Technologies, Inc, 2022).

### 2.8 Searching the spectral database for organic compounds (SDBS)

Spectral matching was performed using the spectral database for organic compounds ( https://sdbs.db.aist.go.jp/SearchInformation.aspx) to identify the probable chemical constituents. Two parameters are set by the database developers to control the quality of the obtained search results: 1) the absorption wavelength matching range (Allowance), expressed as ± number (±1, ±2, etc.); and 2) the transmission percentage. Precise search results are obtained by minimizing both the Allowance and the transmission percentage. The set of IR spectra search criteria depends on the nature of the sample, whether it is crude or pure and the processing method. Low values for the Allowance range and transmission percentage are generally set for pure compounds ( SDBS, 2025; Coates, 2006). The SDBS search criteria are presented in a supplementary file (Supplement 1).

The PubChem database ( https://pubchem.ncbi.nlm.nih.gov/) was employed to identify the basic chemical information and functions of the identified compounds. Additionally, some of the biological activities of the suggested compounds were gathered by searching the published literature.

### 2.9 Statistical analysis

The results of the blood glucose concentration were analyzed using the Statistical Package for Social Sciences (SPSS) version 20. The mean values of the blood glucose concentration of the different study groups were compared using the Analysis of Variance (ANOVA) test, with significant variation determined at a level of p-value ≤ 0.05.

## 3. Results

To enhance the clarity of this article, the results section is divided into three parts: IR Spectral Analysis, Compound Identification, and GTT Results, highlighting the bioactivity of the water and ethanol extracts.

### 3.1 IR Spectral analysis

The analysis of the infrared (IR) spectra provided insight into the chemical composition of the extracts.

**Figure 1.**
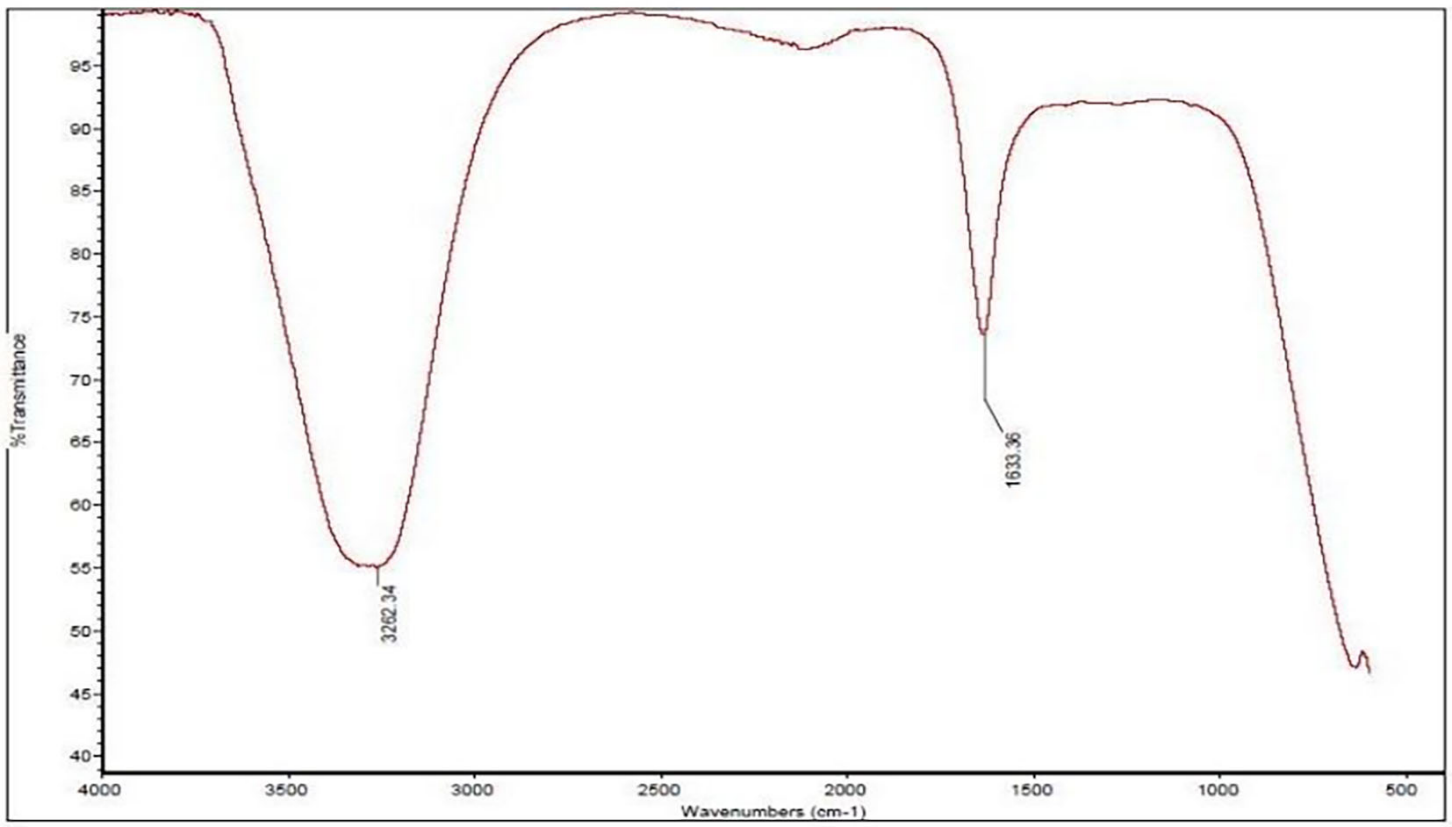
Shows the IR spectrum of the water extract, highlighting two distinct peaks.

In the case of the water extract, two primary IR peaks were identified at 1633.36 cm ^−1^ and 3262.34 cm ^−1^ [ Figure 1]. The peak at 1633.36 cm ^−1^ is correlated with C=C stretching or bending vibrations, indicating the presence of carbonyl compounds or conjugated systems, whereas the peak at 3262.34 cm ^−1^ indicates hydroxyl groups (-OH), proposing a rich content of phenolic compounds and other alcohols ( Coates, 2006).

**Figure 2.**
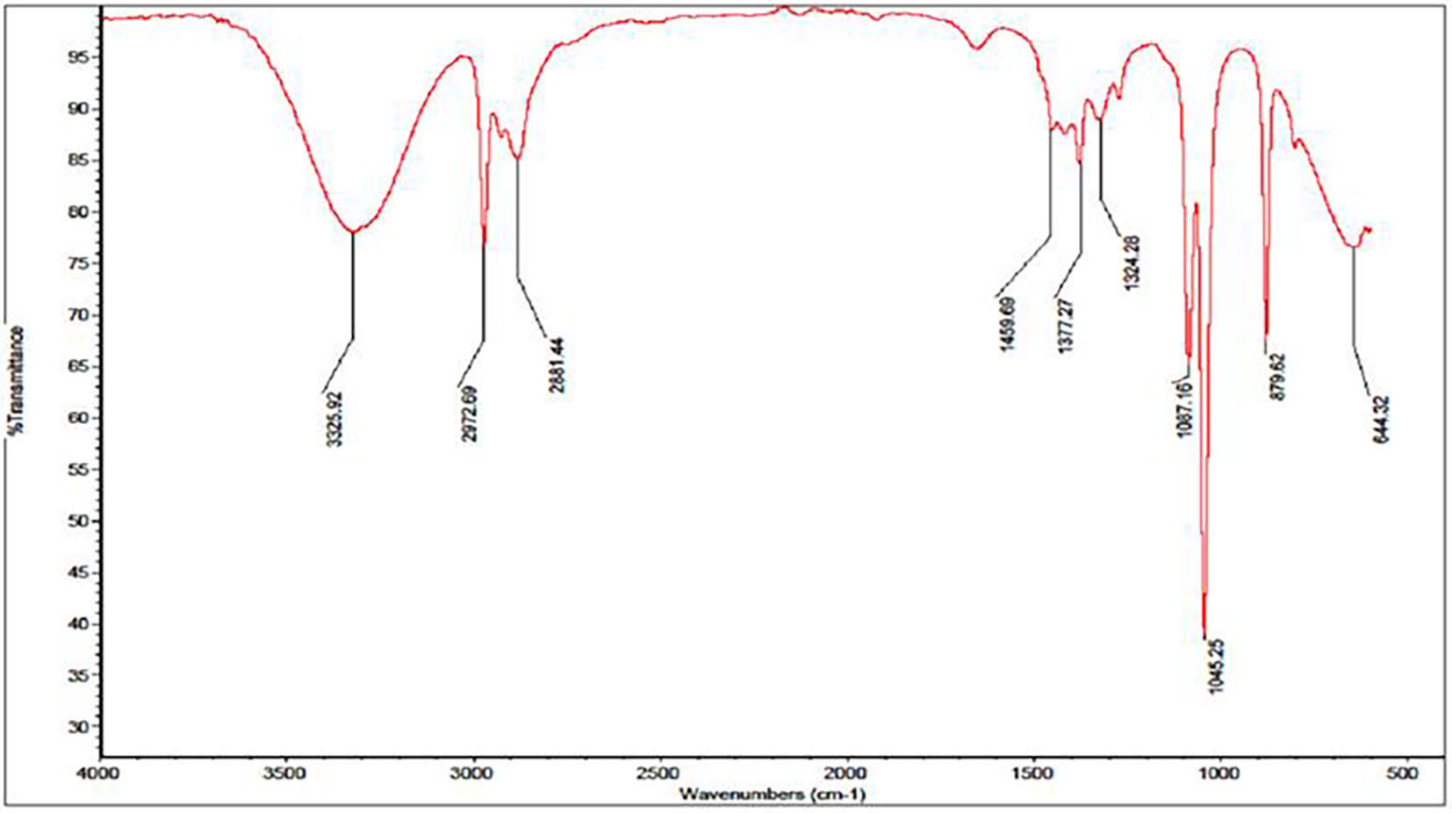
Displays the IR spectrum of the ethanol extract emphasizing 10 noticeable peaks.

For the ethanol extracts, a broader range of IR peaks was observed, specifically at 644.32 cm ^−1^, 879.62 cm ^−1^, 1045.25 cm ^−1^, 1087.16 cm ^−1^, 1324.28 cm ^−1^, 1377.27 cm ^−1^, 1459.69 cm ^−1^, 2881.44 cm ^−1^, 2972.69 cm ^−1^, and 3325.92 cm ^−1^ [ Figure 2]. These peaks provide details about various functional groups present in the ethanol extracts. Notably, the peaks around 1045.25 cm ^−1^ and 1087.16 cm ^−1^ are characteristic of C–O stretching vibrations, often associated with sugar derivatives and alcohols. Additionally, the peaks at 2881.44 cm ^−1^ and 2972.69 cm ^−1^ relate to C–H stretching, suggesting the presence of aliphatic hydrocarbons, while the peak at 3325.92 cm ^−1^ indicates the presence of hydroxyl groups ( Coates, 2006).

This comparative analysis highlights the differences in the chemical profiles of the water and ethanol extracts, which may contribute to their varying biological activities as observed in the glucose tolerance test.

### 3.2 Compound identification

As previously mentioned, the identification of the corresponding compounds for the IR peaks was accomplished by searching the spectral database of organic compounds (SDBS). The chemical structures and their functions were further elucidated using the PubChem database and relevant literature.

In the case of the water extract, the two IR peaks identified were found to correspond to a total of 39 compounds within the SDBS. A comprehensive list of these compounds is provided in ( Table 1- Table 7) (Supplement 2).

**Table 1.** The sugars and their derivatives in the water and ethanol extract of the workers and drones of *Apis mellifera jemenitica.*

	Class	Compound and formula	Chemical structure	Biological activity	Extract	Pubchem ID
1	Sugars and their derivatives	6-deoxy-beta-L-galactopyranose (L-Fucose) C _6_H _12_O _5_	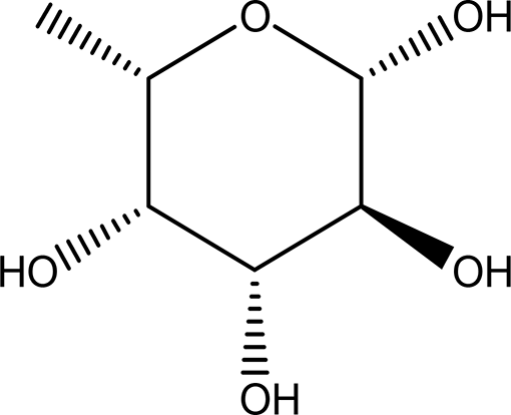	Antitumor Anti-aging Relief of intestinal disease Component in skin care products Emulsifier in food industry ( Wang et al., 2024; Adhikari et al., 2022; Garber et al., 2021; Al-Baarri et al., 2018; 21. Fiume et al., 2019).	Water and ethanol	Pubchem: 444863 SDBS:32641
		6- deoxy-D- galactose (D- Fucose) C _6_H _12_O _5_	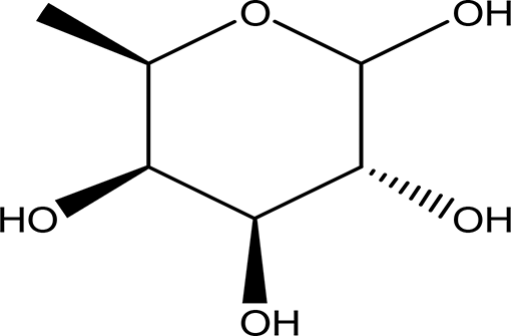	Anti- human acrosin which causes male infertility ( National Center for Biotechnology Information, 2024a; Klemm et al., 1991).	Ethanol	Pubchem: 444200 SDBS:2561
		(S)-1,2-O-(2,2,2-trichloroethylidene)-alpha-D-glucofuranose C _8_H _11_Cl _3_O _6_	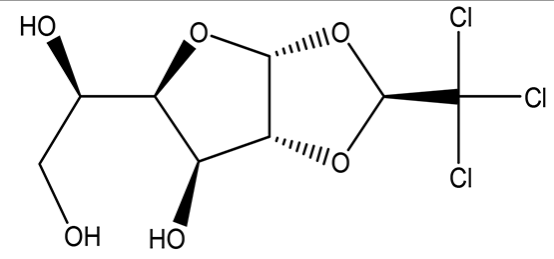	Sedative Anesthetic in animal experiments Induction of drowsiness and sleep ( National Center for Biotechnology Information, 2024b).	Ethanol	Pubchem: 5284343 SDBS:15171
		methyl 2,3,4-tri-O-acetyl-1-deoxy-1-(propoxy (thiocarbonyl))amino-beta-D-glucopyranuronate C _17_H _25_NO _10_S	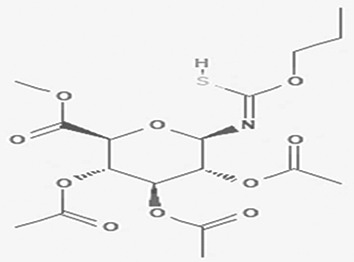	May increase bone matrix deposition ( Nagaoka et al., 2012).	Ethanol	Pubchem: 273075659 SDBS:32735

The only compound which is reported in both the water and ethanol extracts is the L- fucose.

**Table 2.** The phenolic compounds in the water and ethanol extract of the workers and drones of *Apis mellifera jemenitica.*

	Class	Compound	Chemical structure	Biological activity	Extract	ID
2	Phenolic compounds	2,4,6-trimethylpyridinium p-toleunesulfonate C _15_H _19_NO _3_S	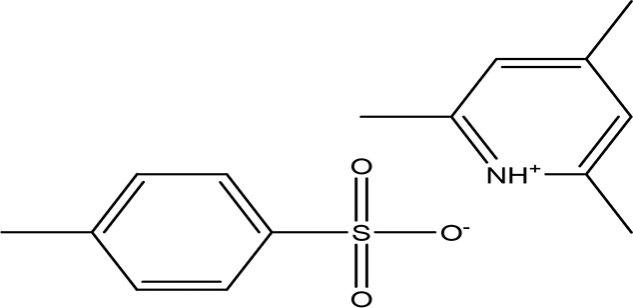	It is used for chemical synthesis ( Chemical book, 2023).	Water	Pubchem: NA SDBS:18269
		2,4- dihydroxybenzoic acid C _7_H _6_O _4_	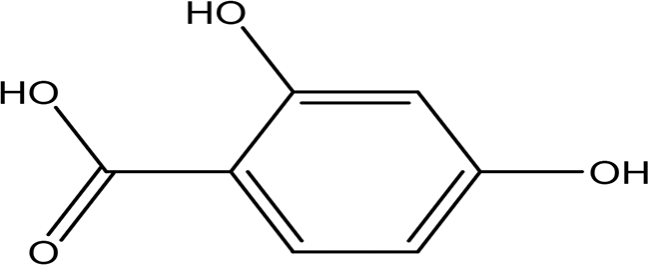	Plays a role in plant immunity Antioxidant and antimicrobial ( Lu et al., 2024; Kalinowska et al., 2021).	Water	Pubchem: 1491 SDBS:3084
		2,3-naphthalenediol C _10_H _8_O _2_	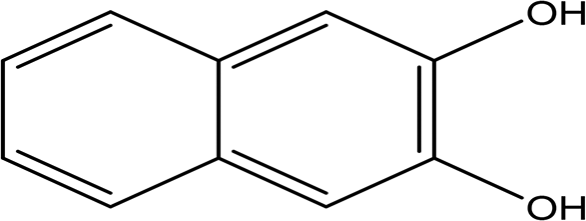	Antioxidant, antiplatelet aggregation, anti-inflammatory, antimicrobial and anti-protozoa ( Ibrahim and Mohamed, 2016).	Water	Pubchem: 7091 SDBS:1626
		6-methyl-2-benzothiazolamine C _8_H _8_N _2_S	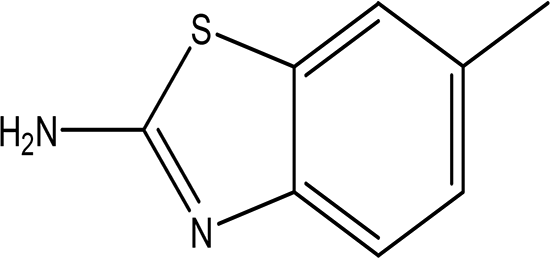	Anti-tubercular, antimicrobial, anti-inflammatory, anti-convulsion, anti-diabetic and anticancer ( Ali and Siddiqui, 2013; Dhadda et al., 2021).	Water	Pubchem: 17335 SDBS: 3579
		3-amino-4-hydroxybenzenesulfonic acid C _6_H _7_NO _4_S	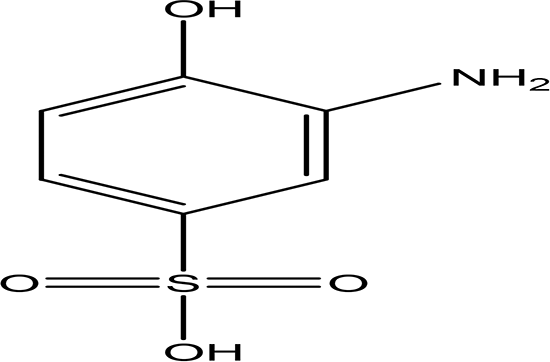	Anti- acute myeloid leukemia inhibitor of some enzymes antagonist and agonist of some pathways ( National Center for Biotechnology Information, 2024f).	Water	Pubchem: 7385 SDBS: 10121
		5-(p-aminophenyl)-2-thiazolamine C _9_H _9_N _3_S	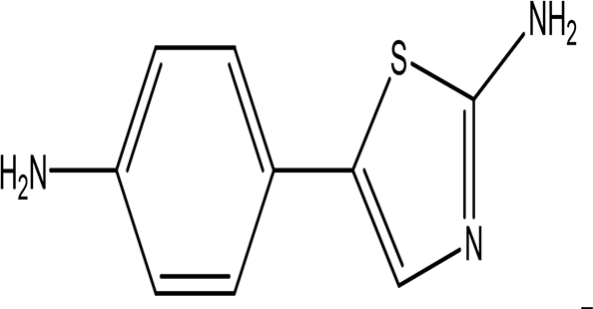	Collectively, the amino phenol and the thiazoleamine have antimicrobial and anti-diabetic activities ( Rafique et al., 2022; Ali and Sayed, 2021).	Water	Pubchem: 605642 SDBS:32115
		3'-hydroxy-2'-acetonaphthone C _12_H _10_O _2_	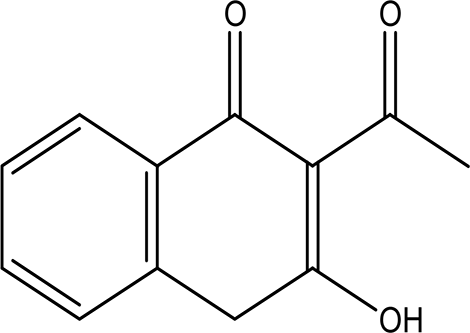	It may act as antimicrobial and anti-convulsion ( Karakurt et al., 2010; National Center for Advancing Translational Sciences, 2020).	Water	Pubchem: NA SDBS: 28941
		4-nitro-2-(trifluoromethyl)aniline C _7_H _5_F _3_N _2_O _2_	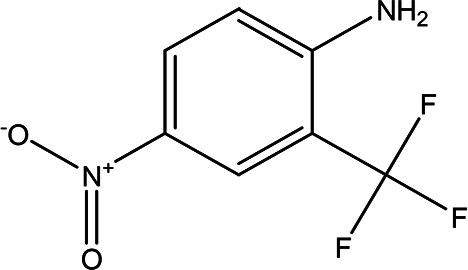	It is used for the synthesis of monoazo dyes ( Dickey et al., 1953). Anti-tuberculosis, antiviral, anticancer and antidepressant ( Nair et al., 2022).	Water	Pubchem: 67128 SDBS:3261
		5,6-dihydro-4H-benzo(6,7)cyclohepta(1,2-d)thiazol-2-amine hydrobromide C _12_H _12_N _2_S HBr	NA	Anti-angiogenesis ( Bhat et al., 2013).	Water	Pubchem: NA SDBS:26013
		O-(N-(dimethylcarbamoylmethyl)acetamido)-N-N-dimethylbenzamide C _15_H _21_N _3_O _3_	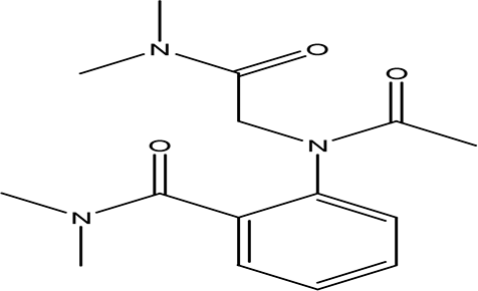	Anticancer, Anti-epilepsy, antiviral, anti-Alzheimer and urease inhibitor ( Ghosh and Brindisi, 2019; Matošević and Bosak, 2020; Ahmad et al., 2023a).	Water	Pubchem: 600891 SDBS:32336
		8-cyano-3,3-diphenyl-3,3a-dihydrocyclohepta(b)furan-2-one C _22_H _15_NO _2_	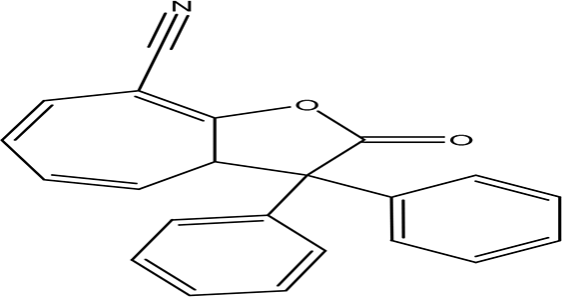	Anti-tumor, antimicrobial, antioxidant and anti-inflammatory ( Miao et al., 2019).	**Water**	Pubchem: 275779763 SDBS:30670
		2-ethylthio (thiocarbonyl)amino-phenylpropionic acid cyclohexylamine salt C _12_H _15_NO _2_S _2_ C _6_H _13_N	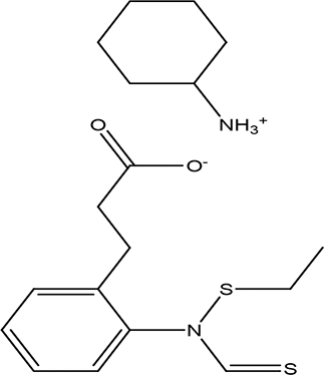	**Phenylprpoanoid derivatives** Antimicrobial, antioxidant, anti-inflammatory, antidiabetic and anticancer ( Neelam et al., 2020). **Cyclohexylamine derivatives** Analgesic activity and decrease the the motor activity ( Glushkov et al., 2006).	**Water**	Pubchem: NA SDBS:29929
		Tiropramide hydrochloride C _28_H _41_N _3_O _3_ HCl	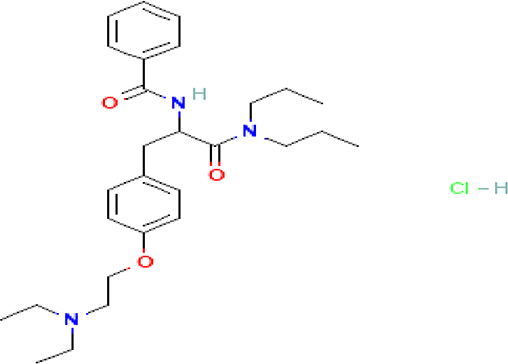	Antispasmodic for hepatobiliary and urinary tract diseases ( National Center for Biotechnology Information, 2024h, Lee et al., 2013; Center for Advancing Translational Sciences, 2024).	**Water**	Pubchem: 134448 SDBS: 53486
		2-methyl-1,2,3,4-tetrahydro-2-naphthol C _11_H _14_O	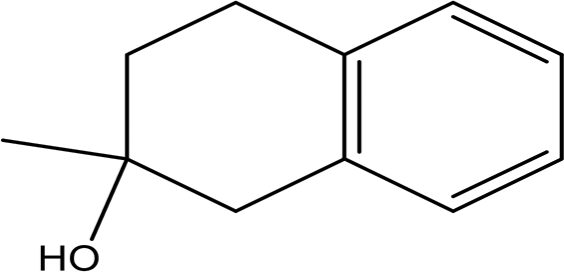	Antioxidant and inhibitor of acetylcholinesterase ( Erdoğan et al., 2021).	Ethanol	Pubchem: NA SDBS: 31692
		(4aalpha, 7alpha, 9alpha, 9aalpha)-9-9a-epoxy-1,1,41,7-tetramethyl-2,3,4,4a,5,6,7,8,9,9a-decahydro-1H-benzocyclohepten-7-ol C _15_H _26_O _2_	NA	As a benzocycloheptane derivative it can act as antihistamine and anti-hepatoma ( Abounassif et al., 2005; Liang et al., 2020).	Ethanol	Pubchem: NA SDBS: 32776
		ethyl p-((2-chloroethoxy) carbonylamino) benzoate C _12_H _14_CINO _4_	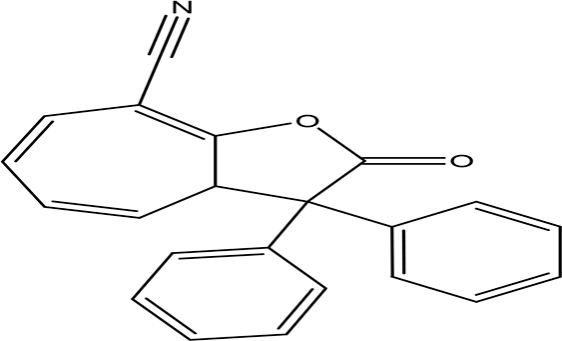	benzoate derivatives have antioxidant, anticancer, antimicrobial, anti-Alzheimer and they are used as pesticides Zou et al., 2019; Haroon et al., 2023; Lee et al., 2023; Elabasy et al., 2019).	Ethanol	Pubchem: 273078333 SDBS: 33657
		Lasalocid Sodium Salt C _34_H _53_NaO _8_	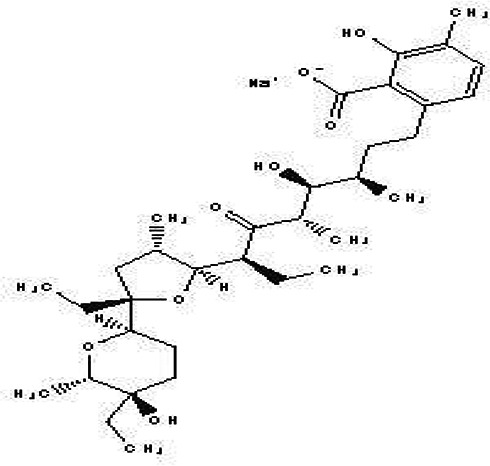	Veterinary antimicrobial and ionophore ( National Center for Biotechnology Information, 2024i).	Ethanol	Pubchem: 6426773 SDBS: 21356
		methyl 4-(3,5-dichloro-4-methoxyphenyl)-3-ethyl-1-pyrazoline-3-carboxylate C _14_H _16_Cl _2_N _2_O _3_	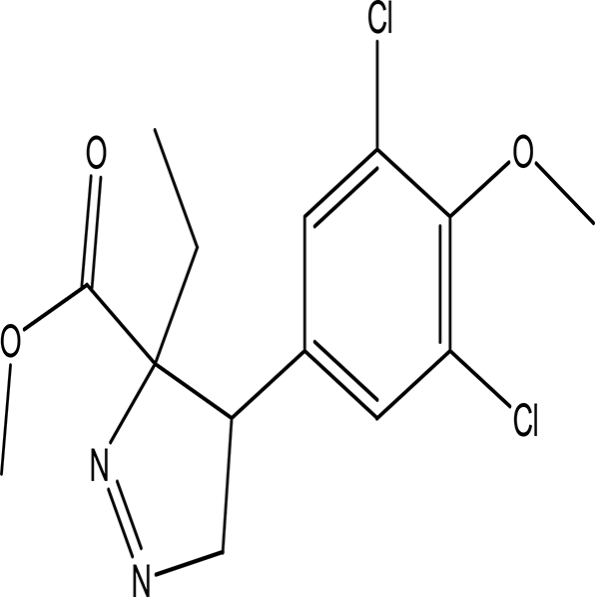	Methoxyphenyl dervitives act as anti food spoilage bacteria and antioxidant ( Orlo et al., 2021; Ahmad et al., 2023b). Pyrazole derivatives exhibit a wide range of pharmacological activities including anticancer, antimicrobial, antioxidant, anti-obesity and antihypertension ( Karrouchi et al., 2018; Hassan, 2013).	Ethanol	Pubchem: 274969331 SDBS: 39016
		2',7'-dihydroxyspiro (isobenzofuran-1(3H),9'-(9H)xanthen)-3-one C _20_H _12_O _5_	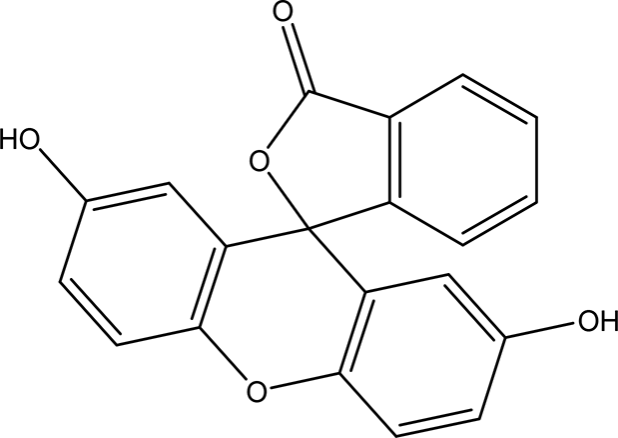	Antitumor, antibacterial, antiviral and antioxidant ( Miao et al., 2019). Anticancer, anti-proliferative ( Zukić et al., 2020).	Ethanol	Pubchem: 625532 SDBS: 35197

The phenolic compounds are the dominant natural products in the water and ethanol extracts of whole body extracts of *Apis mellifera jementica.* The hypoglycemic activity of the water extract may be due to its content of phenolic compounds.

**Table 3.** The alkaloids in the water and ethanol extract of the drones and workers of *Apis mellifera jemenitica.*

	Class	Compound	Chemical structure	Biological activity	Extract	Pubchem ID
3	Alkaloids	2-(5-methyl-3-pyrroly) piperidine hydrochloride C _10_H _16_N _2_ HCl	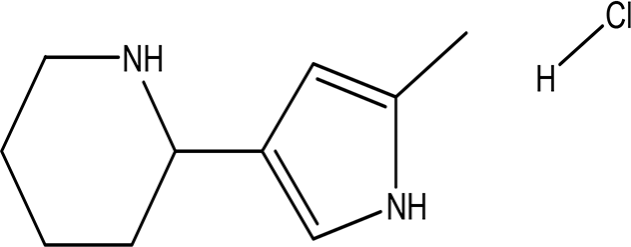	Anticancer, antimicrobial, anti-Alzheimer, antioxidant, anti-neuropathic pain, anti-hypertension, anti-asthma, anti-inflammation ( Frolov and Vereshchagin, 2023; Abdelshaheed et al., 2021).	Water	Pubchem: NA SDBS: 37497
		3-methyl-4-oxo-3,4-dihydro-1-phthalazinecarbohydrazide C _10_H _10_N _4_O _2_	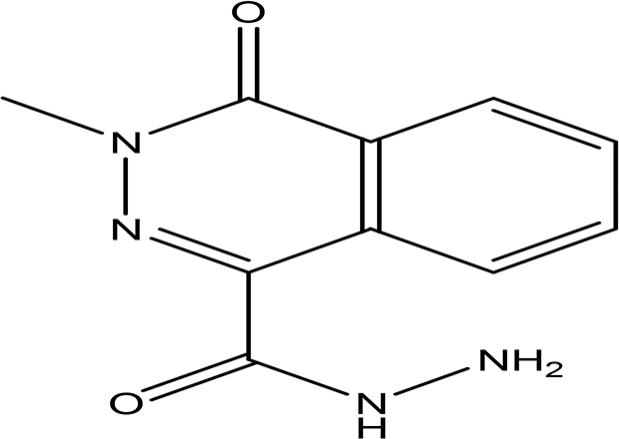	**Phthalazine derivatives** Anticancer, anti-diabetes, anti-hypertension, anti-microbes, anti-depression and they have analgesic activity ( Sangshetti et al., 2019). **Carbohydrazide derivatives** Antibacterial, antifungal, anti-inflammatory and anti-tuberculosis ( Onyeyilim et al., 2022).	Water	Pubchem: 604579 SDBS: 26308
		N-(5-chloro-6-oxo-1-phenyl-1,6-dihydro-4-pyridazinyl)acetamide (Alkaloid and phenolic) C _12_H _10_ClN _3_O _2_	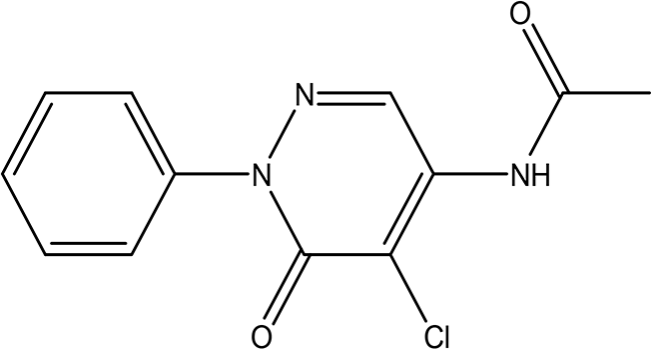	**Pyridazine derivatives** Antitumor, antibacterial and antifungal ( He et al., 2021; Butnariu and Mangalagiu, 2009). **Acetamide derivatives** Antioxidant and anti-inflammatory ( Autore et al., 2010).	Water	Pubchem: 614997 SDBS: 34810
		2-hydrazino-3,5,6,7-tetrahydrocyclopentapyrimidin-4-one C _7_H _10_N _4_O	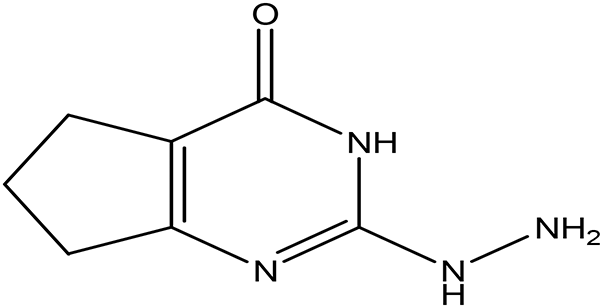	**Hydrazine derivatives** Carbonic anhydrase inhibitors Goff and Ouazzani, 2014; Shirinzadeh and Dilek, 2020). **Cyclopentapyrimidin-4-one derivatives** Phosphodiesterase 10A inhibitors ( Al-Nema et al., 2022; Bhawale et al., 2023).	Water	Pubchem: 273075486 SDBS:32423
		3'-(9-methyl-9H-pyridazino(3,4-b)indol-3-yl)acetanilide C _19_H _16_N _4_O	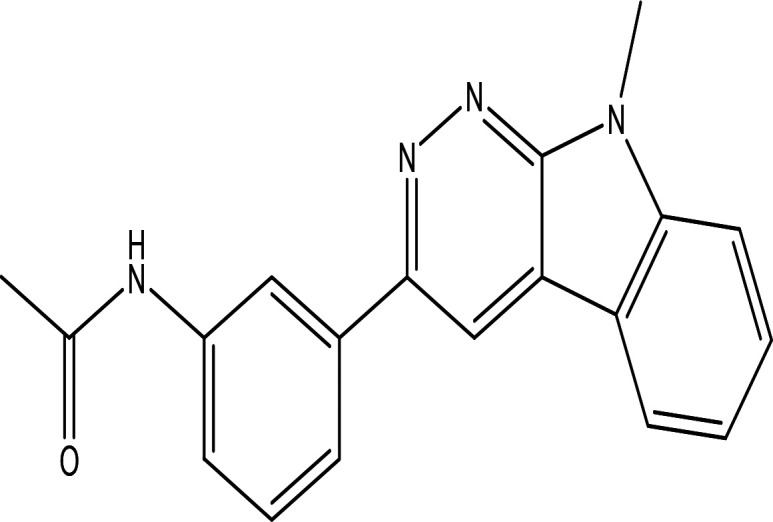	**Pyridazine derivatives** Antitumor, antibacterial and antifungal ( He et al., 2021; Butnariu and Mangalagiu, 2009). **Indole derivatives** Antimicrobial, anti-malarial, anti-diabetes, anti-inflammatory and anti-tuberculosis ( Kumar and Ritika, 2020). **Acetanilide derivatives** Analgesic, anti-inflammatory, antipyretic, antioxidant, anticonvulsant, antimicrobial, an-ti-cancer, anti-hyperglycaemia and antimalarial ( Singh et al., 2019).	Water	Pubchem: 273078537 SDBS:34070
		Cytochalasin E C _28_H _33_NO _7_	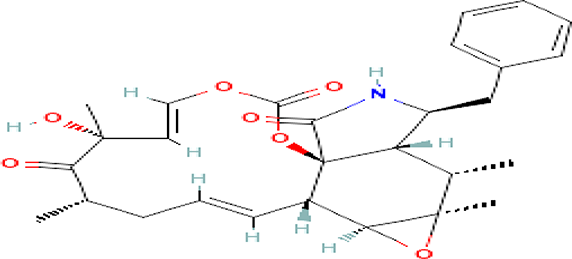	Inhibitor of angiogenesis and increases the sensitivity of lung cancer to chemotherapy ( National Center for Biotechnology Information, 2024j; Takanezawa et al., 2018).	Water	Pubchem: 5458385 SDBS:13826
		1,4'-bipiperidine C _10_H _20_N _2_	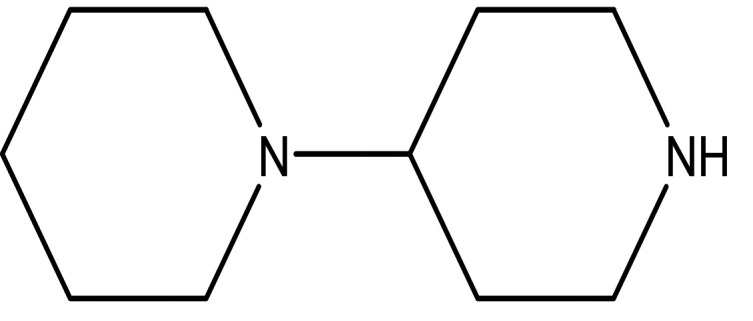	Anticancer, antimicrobial, anti-Alzheimer, antioxidant, anti-neuropathic pain, anti-hypertension, anti-asthma, anti-inflammation ( Frolov and Vereshchagin, 2023; Abdelshaheed et al., 2021).	Ethanol	Pubchem: 78607 SDBS:22415

The water extract was rich in alkaloids while only one alkaloid was reported in the ethanol extract. The blood glucose lowering activity may be attributed to its alkaloid content.

**Table 4.** The quinones in the water extract of the drones and workers of *Apis mellifera jemenitica.*

	Class	Compound	Chemical structure	Biological activity	Extract	ID
4	Quinones	4,5-dianilino-O-benzoquinone C _18_H _14_N _2_O _2_	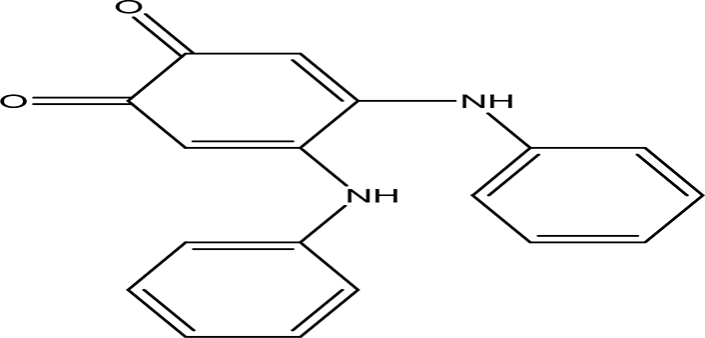	Antimalarial, anti-Alzheimer, antiviral, antifungal, antibacterial and antitumor ( Huang et al., 1993; Zhang et al., 2021).	Water	Pubchem: 274966116 SDBS:34956
		1-amino-2-bromo-4-hydroxyanthraquinone C _14_H _8_BrNO _3_	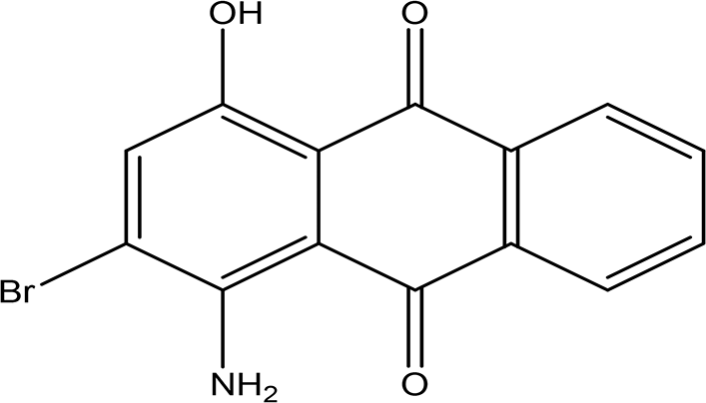	Antioxidant, anticancer, anti-inflammation and anti-aging, hepato-protective and neuro-protective ( Zhao and Zheng, 2023).	Water	Pubchem: 8320 SDBS:18790
		2,5-bis(2-hydroxypropylamino)-p-benzoquinone C _12_H _18_N _2_O _4_	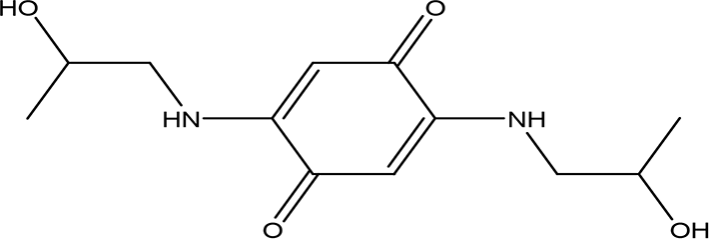	Antimalarial, anti-Alzheimer, antiviral, antifungal, antibacterial and antitumor ( Zhao and Zheng, 2023).	Water	Pubchem: 620240 SDBS:25209
		2,5,6-trihydroxy-1,4-naphthoquinone C _10_H _6_O _5_	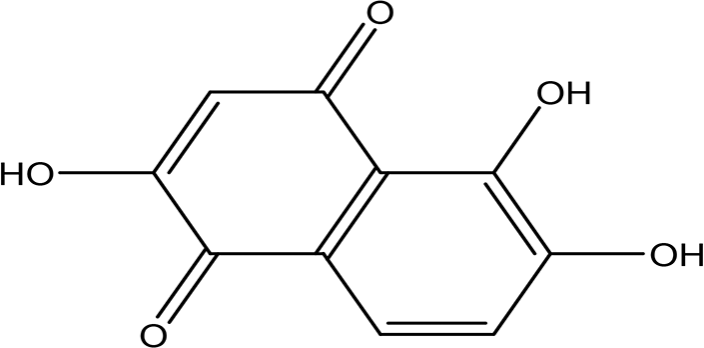	Anticancer, antibacterial, cytotixic, anti-infammatory and antioxidant ( Nematollahi et al., 2012; Li et al., 2018).	Water	Pubchem: 273072755 SDBS:31333

Quinones are specific to the water extract of the whole body Apis melliferal jementica.

**Table 5.** The dipeptides in the water extract of the drones and workers of *Apis mellifera jemenitica.*

	Class	Compound	Chemical structure	Biological activity	Extract	ID
5	Dipeptides	DL-alanyl-L-phenylalanine C _12_H _16_N _2_O _3_	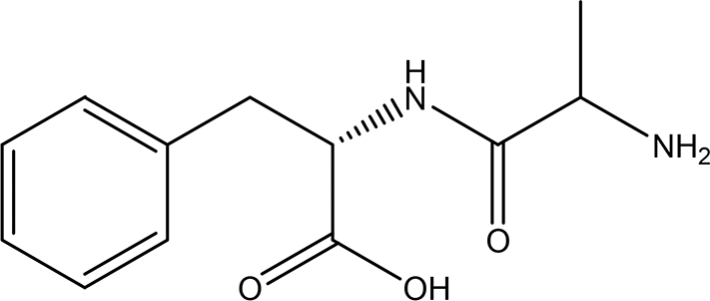	**ACE and Renin inhibitor** Anti-hypertension **DPP IV inhibitor** Anti-hyperglycemia (anti-diabetes) ( Gallego et al., 2019; Messerli et al., 2018; Deacon, 2020; Fisher and Meagher, 2011).	Water	Pubchem: 2080 SDBS:4845
		n-isovaleryl-L-alanine anilide C _14_H _20_N _2_O _2_	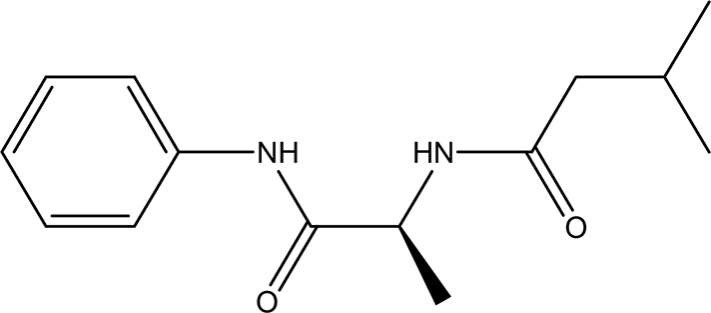	**Anilide derivatives with glycine** Anti-convulsions of Epilepsy ( Soyer et al., 2013). Anti-hypertension, anti-diabetes and antioxidant ( Gallego et al., 2019).	Water	Pubchem: 564405 SDBS:32067

Dipeptide are reported to have anti-diabetes activity as stated my some of previously published articles.

**Table 6.** The amino acids derivatives in the water extract of the drones and workers of *Apis mellifera jemenitica.*

	Class	Compound	Chemical structure	Biological activity	Extract	ID
6	Amino acid derivatives	L-glutamic acid 5-hydrazide C _5_H _11_N _3_O _3_ H _2_O	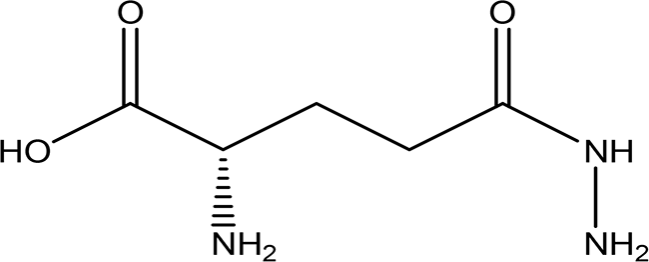	It has strong mutagenic activity on E coli ( Maeda et al., 2021). Ii is involved in the anabolism of fosfazinomycin and kanamycin ( Wang et al., 2018).	Water	Pubchem: 92165 SDBS:29556
		(R)-noradrenaline C _8_H _11_NO _3_	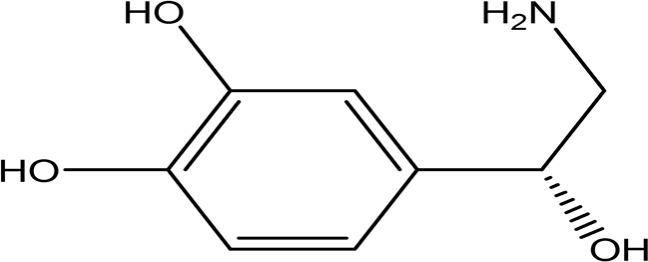	It is a neurotransmitter and vasoconstrictor used for the treatment of hypotension ( Smith and Maani, 2024).	Water	Pubchem: 439260 SDBS:3536
		N (alpha)-benzoyl-DL-arginine-p-nitroanilide hydrochloride C _19_H _22_N _6_O _4_ HCl	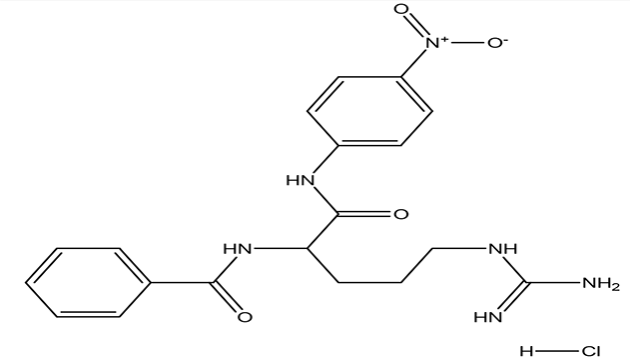	Enhance proteolytic activity of plasmin and trypsin ( Christensen and, Müllertz, 1974; Dulay et al., 2005).	Water	Pubchem: NA SDBS:12532
		1-nitroguanidine CH _4_N _4_O _2_	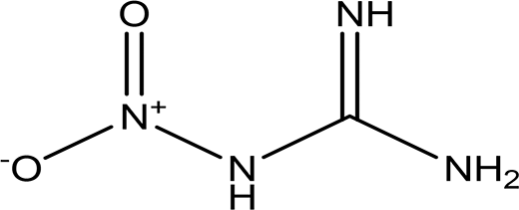	Component of insecticides and explosives ( National Center for Biotechnology Information, 2024k).	Water	Pubchem: 86287517 SDBS:3695

The amino acids derivatives are specific to the water extracts with variable functions for the honeybee. Presence of 1-nitroguanidine may be due to environmental pollution.

**Table 7.** The organometallic compounds and pesticides in the water extract of the drones and workers of *Apis mellifera jemenitica.*

	Class	Compound	Chemical structure	Biological activity	Extract	ID
7	Organometallic compounds	Pentaammine (chloroacetato) cobalt (III)diperchlorate C _2_H _17_Cl _3_CoN _5_O _10_	NA	Used in synthesis of explosive material ( Ilyushin et al., 2010).	Water	Pubchem: NA SDBS:35464
		Potassium diaquabis (malonato) manganite (III) C _6_H _8_KMnO _10_ 2H _2_O	NA	synthetic Inorganic compound with magnetic activity ( Delgado et al., 2006).	Water	Pubchem: NA SDBS:26382
		alpha-chloralose C _8_H _11_Cl _3_O _6_	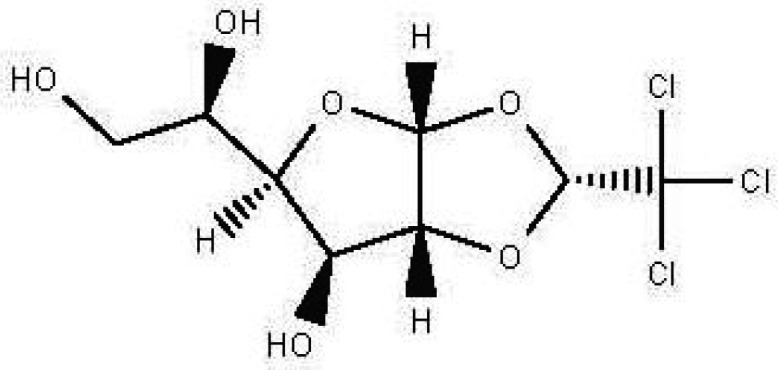	Pesticide, anesthetic, hypnotics and sedatives ( National Center for Biotechnology Information, 2024 ^l^).	Ethanol	Pubchem: 7057995 SDBS:3495

The presence of the organometallic compounds may be referred to environmental pollution.

For the ethanol extract, the spectral analysis revealed that its IR peaks corresponded to 12 compounds, as detailed in ( Table 1- Table 7) (Supplement 3).

The compounds identified in both extracts were categorized into various groups based on their chemical nature and biological potential. The classifications include: sugars and sugar derivatives ( Table 1), phenolic compounds ( Table 2), alkaloids ( Table 3), quinones ( Table 4), dipeptides ( Table 5), amino acid derivatives ( Table 6) and organometallic compounds and pesticides ( Table 7).

This classification not only aids in understanding the chemical complexity of the extracts but also provides insight into their potential biological activities, which may be relevant for their applications in medicinal and therapeutic contexts.

### 3.3 GTT results

*3.3.1 Patterns of GTT curves*

**Figure 3.**
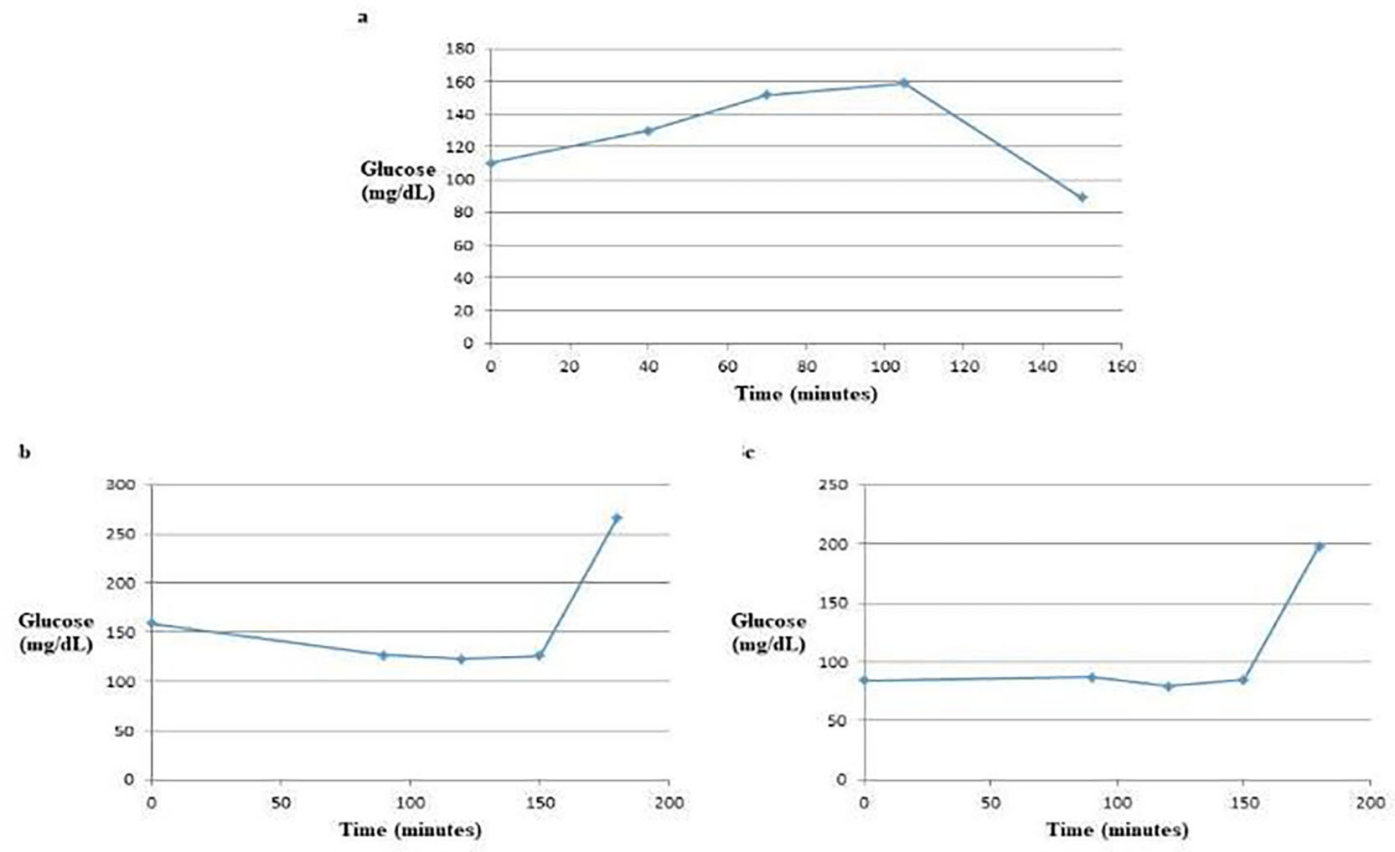
The GTT Curves of glucose alone (a), glucose with water extract (b) and glucose with ethanol extract (c). The water extract altered the GTT curve from a convex shape to a concave shape, indicating a significant effect on blood glucose levels. In contrast, the ethanol extract demonstrated a more variable response, showing a slight increase, followed by a slight decrease, and then a subsequent increase in blood glucose levels. This suggests different mechanisms of action or efficacy between the two extracts in influencing glucose tolerance.

The glucose tolerance test (GTT) curve for glucose alone exhibited two distinct phases: an initial increase followed by a decrease. In contrast, the GTT curve for glucose mixed with the water extract demonstrated a decrease initially followed by an increase. The GTT curve involving glucose mixed with the ethanol extract was more complex, displaying three phases: a slight increase, a slight decrease, and then a subsequent increase [ Figure 3]. These variations in the GTT curves can likely be attributed to the different constituents present in the water and ethanol extracts of the *A. m. jemenitica* drones and workers.

*3.3.2 Comparison of the blood glucose results*

The glucose concentration in the blood samples was measured from the rabbits at two distinct time points: at zero time (fasting) and after breaking the fast at intervals of 90, 120, 150, and 180 minutes. Blood samples were collected from three different groups involved in the glucose tolerance test (GTT): a control group that received glucose alone, a group that received glucose combined with honeybee water extract, and another group that received glucose combined with honeybee ethanol extract. This setup aimed to assess the effects of the honeybee extracts on glucose metabolism compared to the control group. The results of the blood glucose concentration in the three groups and in the different time intervals are presented in Table 8.

**Table 8.** Blood glucose concentration (mg/dL) of the GTT experiments.

Time of blood sample	GTT	Mean	SD	Minimum	Maximum
**Fasting**	Control	97.50*$	17.68	85.00	110.00
	Glucose+ water extract	136.50$@	31.82	114.00	159.00
	Glucose + ethanol extract	97.00$	18.38	84.00	110.00
After 90 minutes	Control	114.50#$	21.92	99.00	130.00
	Glucose+ water extract	120.50$	9.19	114.00	127.00
	Glucose+ ethanol extract	90.00$	4.24	87.00	93.00
After 120 minutes	Control	173.00*#$&@	29.70	152.00	194.00
	Glucose+ water extract	123.50$	.71	123.00	124.00
	Glucose + ethanol extract	82.50$	4.95	79.00	86.00
After 150 minutes	Control	100.50$&	16.26	89.00	112.00
	Glucose+ water extract	115.50$	14.85	105.00	126.00
	Glucose + ethanol extract	124.50	27.58	105.00	144.00
After 180 minutes	Control	87.50$@	10.61	80.00	95.00
	Glucose+ water extract	236.00*#&@	43.84	205.00	267.00
	Glucose + ethanol extract	192.00*#&@	7.07	187.00	197.00

The glucose concentration in the GTT control group was significantly different from its concentration in the other groups. This significant variation (p-value ≤ 0.05) is indicated by five symbols: *, #, $, &, and @. The ANOVA analysis results, including the p-values, are attached to this article as a supplementary file (Supplement.4).

## 4. Discussion

This study can be seen as the first to report the presence of bioactive natural products such as polyphenols, alkaloids, and quinones in the whole body constituents of *A. m. jemenitica* drones and workers. Previously published articles have primarily focused on the nutritional value of honeybees and their larvae and pupae, emphasizing their content of amino acids, fatty acids, proteins, minerals, and vitamins ( Guiné et al., 2022; Ghosh et al., 2016).

### 4.1 Chemical composition of the water and ethanol extracts

*4.1.1 Sugars and their derivatives*

The water extract contained one sugar, L-fucose, whereas the ethanol extract contained two sugars, L-fucose and D-fucose, along with two sugar derivatives. ((S)-1,2-O-(2,2,2-trichloroethylidene)-alpha-D-glucofuranose and methyl 2,3,4-tri-O-acetyl-1-deoxy-1-(propoxy (thiocarbonyl))amino-beta-D-glucopyranuronate).

4.1.1.1 L-fucose (6-deoxy-beta-L-galactopyranose)

Fucose was identified in both the water and ethanol extracts, and it stands out as the only deoxy monosaccharide present in mammals in the L-form, while most other monosaccharides exist in their D-form. Known as 6-deoxy-beta-L-galactose, fucose typically adopts a pyranose configuration and exhibits a crystalline white appearance, with a molecular formula of C _6_H _12_O _5_ ( Table 1 and Table 2). This compound is commonly found at the terminal positions of oligosaccharides, polysaccharides, and glycolipids. L-fucose is abundant in various organisms, such as brown algae, marine microalgae (which include both green and red algae, as well as diatoms), bacteria, and fungi ( Wang et al., 2024).

In terms of functionality, L-fucose plays significant roles in both medicine and industry. It has been recognized for its antitumor and anti-aging properties, and it can also alleviate intestinal pain. Industrially, L-fucose serves as an emulsifier in the food industry and is incorporated into various skin care products ( Adhikari et al., 2022; Garber et al., 2021; Al-Baarri et al., 2018; Fiume et al., 2019).

4.1.1.2 D-fucose (6- deoxy-D-galactose)

D-fucose is a stereoisomer of L-fucose. Unlike L-fucose, which is more prevalent in human fluids, D-fucose has been reported to exhibit anti-acrosin activity. Acrosin is a sperm protease that plays a crucial role in the fertilization of ova ( National Center for Biotechnology Information, 2024a; Klemm et al., 1991). Notably, D-fucose was identified in the ethanol extract but was not detected in the water extract, as shown in Table 2.

4.1.1.3 (S)-1,2-O-(2,2,2-trichloroethylidene)-alpha-D-glucofuranose (beta-Chloralose)

This derived monosaccharide is detected in the ethanol extract. It was historically used as a sedative drug, but due to its side effects and limited effectiveness, it has been largely replaced by safer and more effective sedatives. Nevertheless, it still finds application as a general anesthetic for animals. In a medical context, it is utilized to relieve psychological excitement by inducing drowsiness and promoting sleep (see Table 2 for further details) ( National Center for Biotechnology Information, 2024b).

4.1.1.4 Methyl 2,3,4-tri-O-acetyl-1-deoxy-1-(propoxy (thiocarbonyl))amino-beta-D-glucopyranuronate

As a glucuronic acid derivative, Methyl 2,3,4-tri-O-acetyl-1-deoxy-1-(propoxy (thiocarbonyl))amino-beta-D-glucopyranuronate may enhance bone matrix deposition and decrease bone resorption. This compound could achieve these effects through the activation of osteoblastic cell differentiation while simultaneously inhibiting osteoclastic cell differentiation. This dual action may contribute to improved bone health and density ( Nagaoka et al., 2012). The Methyl 2,3,4-tri-O-acetyl-1-deoxy-1-(propoxy (thiocarbonyl))amino-beta-D-glucopyranuronate was not detected in the water extract ( Table 2).

*4.1.2 Phenolic compounds*

4.1.2.1 2,4,6-trimethylpyridinium p-toleunesulfonate

The 2,4,6-trimethylpyridinium p-toluenesulfonate was detected in the water extract. It is a white to pale yellow or orange solid with the molecular formula C _15_H _19_NO _3_S. This compound is commonly used in chemical synthesis, serving as a significant reagent in various organic reactions ( Chemical Book. 2023). Its presence in the extracts underlines the varied chemical constituents that could affect the GTT curve.

4.1.2.2 2,4-dihydroxybenzoic acid

2,4-Dihydroxybenzoic acid was identified in the water extract of Apis mellifera jemenitica. It has the molecular formula C _7_H _6_O _4_, appears as a solid, and is characterized by a white color. This compound is commonly found in various plants ( Table 2) and is utilized in the food industry as a flavoring agent. In the plant kingdom, 2,4-dihydroxybenzoic acid is known to play a crucial role in enhancing disease resistance ( National Center for Biotechnology Information, 2024c; Lu et al., 2024). Additionally, it possesses notable antioxidant and antimicrobial properties ( Kalinowska et al., 2021).

4.1.2.3 2,3-naphthalenediol

2,3-naphthalenediol is a white powder with the molecular formula C _10_H _8_O _2_. It is recognized as a human metabolite and is utilized as a hair dyeing material ( Table 2) ( National Center for Biotechnology Information, 2024d). Naphthalenediols exhibit a range of biological activities, including antioxidant, antilplatelet aggregation, anti-inflammatory, antimicrobial, and anti-protozoal effects ( Ibrahim and Mohamed, 2016).

4.1.2.4 6-methyl-2-benzothiazolamine

6-methyl-2-benzothiazolamine was identified in the water extract. It is solid in nature with the molecular formula of C _8_H _8_N _2_S ( Table.2) ( National Center for Biotechnology Information, 2024e). Benzothiazole derivatives are proven to exhibit various biological activities, including anticancer, anti-tuberculosis, antimicrobial, anti-inflammatory, anti-convulsant, and anti-diabetic properties ( Ali and Siddiqui, 2013; Dhadda et al., 2021).

4.1.2.5 3-amino-4-hydroxybenzenesulfonic acid

The compound mentioned is a brown solid with the molecular formula C _6_H _7_NO _4_S, identified as 3-amino-4-hydroxybenzenesulfonic acid ( Table.2) ( National Center for Biotechnology Information, 2024f). This compound exhibits inhibitory effects on a variety of enzymes, including Coenzyme A dehydrogenase, Aldehyde dehydrogenase, and Apurinic/apyrimidinic endonuclease. Additionally, it has been reported to have anticancer properties, particularly in the context of leukemia. The compound also acts as an antagonist for certain receptors, specifically retinoid-related orphan receptor gamma, while functioning as an agonist for some signaling pathways, notably the peroxisome proliferator-activated receptor delta signaling pathway ( National Center for Biotechnology Information, 2024f).

4.1.2.6 5-(p-aminophenyl)-2-thiazolamine

5-(p-aminophenyl)-2-thiazolamine is a notable water extract with the molecular formula C _9_H _9_N _3_S. This compound features an aminophenol moiety and is incorporated into nano-pigments used for printing applications ( Table 2) ( National Center for Biotechnology Information, 2024g). In addition to its use in printing, aminophenol derivatives are recognized for their antimicrobial and anti-diabetic activities. Specifically, compounds that possess thiazolamine and aminophenol structures have demonstrated antimicrobial properties, with sulfathiazol being a prominent example ( Rafique et al., 2022; Ali and Sayed, 2021).

4.1.2.7 3’-hydroxy-2’-acetonaphthone

3’-Hydroxy-2’-acetonaphthone has the molecular formula C _12_H _10_O _2_ and has been identified in the water extract of Apis mellifera jemenitica. As a naphthene derivative, acetonaphthone is noted for its potential antimicrobial and anti-convulsant properties, as indicated by Karakurt et al. (2010) and Center for Advancing Translational Sciences (2020).

4.1.2.8 4-nitro-2-(trifluoromethyl)aniline

The compound 4-nitro-2-(trifluoromethyl) aniline, with the molecular formula C _7_H _5_F _3_N _2_O _2_, is primarily utilized in the synthesis of mono-azo dyes ( Dickey et al., 1953). Additionally, trifluoromethyl compounds have shown potential in the treatment of various diseases, serving as effective agents in anti-tuberculosis, antiviral, anticancer, and antidepressant applications ( Nair et al., 2022).

4.1.2.9 5,6-dihydro-4H-benzo(6,7)cyclohepta(1,2-d)thiazol-2-amine hydrobromide

5,6-Dihydro-4H-benzo(6,7)cyclohepta(1,2-d)thiazol-2-amine hydrobromide is classified as a tricyclic thiazole compound. This compound features derivatives of benzene, cycloheptane, and thiazole, making it a unique structure. It has been noted for its biological activity, particularly as an anti-angiogenesis agent, which suggests it may play a role in inhibiting the growth of new blood vessels that can contribute to tumor development and other pathological conditions. Studies exploring its potential therapeutic applications could be of great interest in drug development and cancer treatment ( Bhat et al., 2013).

4.1.2.10 O-(N-(dimethylcarbamoylmethyl)acetamido)-N-N-dimethylbenzamide

O-(N-(dimethylcarbamoylmethyl)acetamido)-N,N-dimethylbenzamide is classified as a urea derivative. Urea derivatives, which include compounds like acetamide and benzamide, are commonly utilized in drug design. Notable examples include glibenclamide and cariprazine, which are used for treating various conditions. These derivatives have applications in the treatment of certain cancers, epilepsy, viral infections such as hepatitis C and HIV, as well as Alzheimer’s disease ( Ghosh and Brindisi, 2019; Matošević and Bosak, 2020). Additionally, compounds containing acetamide and benzamide are known to function as urease inhibitors, highlighting their significance in pharmacological research and potential therapeutic uses ( Ahmad et al., 2023a).

4.1.2.11 8-cyano-3,3-diphenyl-3,3a-dihydrocyclohepta(b)furan-2-one

The eleventh phenolic compound identified in the water extract has the molecular formula C _22_H _15_NO _2_. Recognized as a benzofuran, the compound 8-cyano-3,3-diphenyl-3,3a-dihydrocyclohepta(b)furan-2-one exhibits potential biological activities such as antimicrobial, anti-tumor, anti-inflammatory, and antioxidant properties. This highlights its significance in terms of therapeutic applications and its potential benefits in health and medicine ( Miao et al., 2019).

4.1.2.12 2-ethylthio (thiocarbonyl)amino-phenylpropionic acid cyclohexylamine salt

The twelfth compound of the water extract is composed of phenylpropanoid derivative and cyclohexylamine. Phenylpropanoids are known for their various biological activities, including antimicrobial, antioxidant, anti-inflammatory, anti-diabetic, and anticancer properties. Additionally, they play a protective role for the kidneys, neurons, heart, and liver ( Neelam et al., 2020). Conversely, cyclohexylamine derivatives demonstrate analgesic effects and can result in decreased motor activity ( Glushkov et al., 2006).

4.1.2.13 Tiropramide hydrochloride

Tiropramide hydrochloride is a phenolic compound with the molecular formula C _28_H _42_ClN _3_O _3_. It is well-known for its anti-spasmodic properties and is commonly used in the treatment of various conditions related to the hepatobiliary and urinary tracts, including Irritable Bowel Syndrome (IBS) ( National Center for Biotechnology Information, 2024h, Lee et al., 2013; Center for Advancing Translational Sciences, 2024).

4.1.2.14 2- methyl-1,2,3,4-tetrahydro-2-naphthol

2-Methyl-1,2,3,4-tetrahydro-2-naphthol has the molecular formula C _11_H _14_O. Naphthol derivatives have been shown to act as antioxidants and acetylcholinesterase inhibitors, which are important since acetylcholinesterase is a marker for degenerative neurological diseases. The antioxidant properties of these compounds may provide protective effects against oxidative stress, while the inhibition of acetylcholinesterase suggests potential therapeutic applications in managing neurological disorders ( Erdoğan et al., 2021).

4.1.2.15 (4aalpha, 7alpha, 9alpha, 9aalpha)-9-9a-epoxy-1,1,41,7-tetramethyl-2,3,4,4a,5,6,7,8,9,9a-decahydro-1H-benzocyclohepten-7-ol

As a benzocycloheptane derivative, (4aα, 7aα, 9aα, 9aα)-9-9a-epoxy-1,1,4,7-tetramethyl-2,3,4,4a,5,6,7,8,9,9a-decahydro-1H-benzocyclohepten-7-ol has the potential to exhibit antihistamine activity, in addition to demonstrating effectiveness in killing hepatoma cells ( Abounassif et al., 2005; Liang et al., 2020).

4.1.2.16 Ethyl p-((2-chloroethoxy) carbonylamino) benzoate

Benzoate derivatives are well-known for their diverse biological activities, including functions as local anesthetics, anticancer agents, anti-Alzheimer compounds, and antimicrobial, antioxidant, and anti-inflammatory agents ( Zou et al., 2019; Haroon et al., 2023). Additionally, these derivatives are utilized in agricultural applications as pesticides ( Lee et al., 2023; Elabasy et al., 2019).

4.1.2.17 Lasalocid Sodium Salt

Lasalocid Sodium Salt is a benzoate derivative that contains lasalocid. Its molecular formula is C _34_H _53_NaO _8_. In veterinary medicine, it serves dual functions as an antibacterial agent and an ionophore. As an ionophore, it enhances calcium influx in muscle fibers, which can have various therapeutic effects ( National Center for Biotechnology Information, 2024i).

4.1.2.18 Methyl 4-(3,5-dichloro-4-methoxyphenyl)-3-ethyl-1-pyrazoline-3-carboxylate

The above compound contains two bioactive moieties: methoxyphenyl and pyrazoline. Compounds with methoxyphenol exhibit antibacterial activity against food spoilage bacteria and possess antioxidant properties ( Orlo et al., 2021; Ahmad et al., 2023b). Pyrazole derivatives are well known for their range of pharmacological activities, which include anticancer, anti-inflammatory, antiviral, antifungal, antioxidant, anti-obesity, antidepressant, antipsychotic, and analgesic effects ( Karrouchi et al., 2018; Hassan, 2013).

4.1.2.19 2’,7’-dihydroxyspiro (isobenzofuran-1(3H),9’-(9H)xanthen)-3-one

The nineteenth phenolic compounds contain two bioactive moieties: benzofuran and xanthen-3-one. Benzofuran derivatives are well-known for their antitumor, antiviral, antibacterial, and antioxidant properties ( Miao et al., 2019). On the other hand, xanthen-3-one derivatives exhibit notable anticancer and anti-proliferative activities ( Zukić et al., 2020).

*4.1.3 Alkaloids*

4.1.3.1 2-(5-methyl-3-pyrroly) piperidine hydrochloride

Natural and synthetic piperdines are known for their diverse biological activities, including anticancer, antioxidant, anti-Alzheimer, antimicrobial, and anti-neuropathic pain properties ( Frolov and Vereshchagin, 2023). Additionally, piperidine exhibits effectiveness against hypertension, asthma, and inflammation ( Abdelshaheed et al., 2021).

4.1.3.2 3-methyl-4-oxo-3,4-dihydro-1-phthalazinecarbohydrazide

The compound contains two bioactive moieties: phthalazine and carbohydrazide. Phthalazine derivatives are recognized for their activity against several health issues, including cancer, diabetes, hypertension, microbial infections, and depression, as well as their analgesic properties ( Sangshetti et al., 2019). On the other hand, carbohydrazide derivatives exhibit a range of biological activities, including antibacterial, antifungal, anti-inflammatory, and anti-tuberculosis effects ( Onyeyilim et al., 2022).

4.1.3.3 N-(5-chloro-6-oxo-1-phenyl-1,6-dihydro-4-pyridazinyl)acetamide

The bioactive moieties of this compound are the pyridazine and acetamide. Pyridazine derivatives are reported to possess antitumor ( He et al., 2021), antibacterial, and antifungal properties ( Butnariu and Mangalagiu, 2009). In contrast, acetamide derivatives are known for their antioxidant and anti-inflammatory effects ( Autore et al., 2010).

4.1.3.4 3’-(9-methyl-9H-pyridazino(3,4-b)indol-3-yl)acetanilide

The compound 3’-(9-methyl-9H-pyridazino(3,4-b)indol-3-yl) acetanilide is notable for containing three significant bioactive groups: pyridazine, indole, and acetanilide. Pyridazine derivatives are particularly recognized for their anticancer, antibacterial, and antifungal activities ( He et al., 2021; Butnariu and Mangalagiu, 2009). Indole derivatives, on the other hand, exhibit a wide range of biological activities including antimicrobial, anti-malarial, anti-diabetic, anti-inflammatory, and anti-tuberculosis effects ( Kumar and Ritika, 2020). Furthermore, acetanilide derivatives are known for their diverse therapeutic actions, which encompass analgesic, anti-inflammatory, antipyretic, antioxidant, anticonvulsant, antimicrobial, anticancer, anti-hyperglycemic, and antimalarial activities ( Singh et al., 2019).

4.1.3.5 2-hydrazino-3,5,6,7-tetrahydrocyclopentapyrimidin-4-one

2-Hydrazino-3,5,6,7-tetrahydrocyclopentapyrimidin-4-one is classified as both a hydrazine derivative and a cyclopentapyrimidin-4-one derivative. Hydrazine derivatives are known for their potential as carbonic anhydrase inhibitors. These inhibitors play a significant role in treating a variety of conditions, including glaucoma, edema, obesity, osteoporosis, epilepsy, and certain types of cancer ( Goff and Ouazzani, 2014; Shirinzadeh and Dilek, 2020). On the other hand, cyclopentapyrimidin-4-one derivatives are recognized for their ability to inhibit Phosphodiesterase10A, making them potential therapeutic targets for various neurodegenerative disorders ( Al-Nema et al., 2022; Bhawale et al., 2023).

4.1.3.6 Cytochalasin E

It is an alkaloid drug used for the inhibition of angiogenesis and it increases the sensitivity of lung cancer to medication ( National Center for Biotechnology Information, 2024j; Takanezawa et al., 2018).

4.1.3.7 1,4’-bipiperidine

As noted earlier, piperidine derivatives have demonstrated a range of biological activities, including anticancer, antioxidant, anti-Alzheimer, antimicrobial, anti-neuropathic pain, anti-hypertensive, anti-asthmatic, and anti-inflammatory effects ( Frolov and Vereshchagin, 2023; Abdelshaheed et al., 2021).

*4.1.4 Quinones*

4.1.4.1 4,5-dianilino-O-benzoquinone

4,5-Dianilino-O-benzoquinone is reported to have weak antitumor activity ( Huang et al., 1993). Ortho and para benzoquinones are routinely used for their various therapeutic effects, including antimalarial, anti-Alzheimer, antiviral, antifungal, antibacterial, and antitumor properties ( Zhang et al., 2021).

4.1.4.2 1-amino-2-bromo-4-hydroxyanthraquinone

As an anthraquinone derivative, 1-amino-2-bromo-4-hydroxyanthraquinone may exhibit a range of beneficial pharmacological properties, including antioxidant, anticancer, anti-inflammatory, and anti-aging effects. Additionally, studies suggest that anthraquinone derivatives possess hepatoprotective and neuroprotective activities, contributing to their potential therapeutic applications in various medical fields ( Zhao and Zheng, 2023).

4.1.4.3 2,5-bis(2-hydroxypropylamino)-p-benzoquinone

Benzoquinone derivatives exhibit a range of medicinal properties, making them significant in pharmacology. They have been studied for their antimalarial, anti-Alzheimer, antiviral, antifungal, antibacterial, and antitumor activities ( Zhang et al., 2021).

4.1.4.4 2,5,6-trihydroxy-1,4-naphthoquinone

1,4-naphthoquinones are studied as natural products and have exhibited anticancer and antibacterial activities ( Nematollahi et al., 2012). Additionally, Li et al. (2018) stated that naphthoquinone derivatives possess cytotoxicity, antioxidant, and anti-inflammatory activities.

*4.1.5 Dipeptides*

4.1.5.1 DL-alanyl-L-phenylalanine

Dipeptides containing alanine from dry cured ham have been reported to have the ability to inhibit angiotensin-converting enzyme (ACE) and dipeptidyl peptidase IV (DPP IV), making them potential candidates for anti-hypertension and anti-hyperglycemic therapeutic agents, respectively ( Gallego et al., 2019; Messerli et al., 2018; Deacon, 2020). Dipeptides that include phenylalanine also exhibit inhibition of ACE and renin, further establishing their suitability as anti-hypertensive compounds ( Gallego et al., 2019; Messerli et al., 2018; Fisher and Meagher, 2011). However, our review of the literature reveals a lack of articles documenting the presence of the Ala-Phe (AF) dipeptide in food, biological fluids, or tissues. Consequently, the functional role of the AF sequence has not been previously addressed.

4.1.5.2 n-isovaleryl-L-alanine anilide

Anilide derivatives with amino acids exhibit potential anti-convulsant properties, as seen with glycine anilide derivatives ( Soyer et al., 2013). Additionally, dipeptides that include valine or alanine have been shown to inhibit enzymes such as ACE, renin, and DPP IV, indicating their possible roles in managing hypertension and diabetes. Furthermore, dipeptides containing valine residues are noted for their antioxidant activity ( Gallego et al., 2019).

*4.1.6 Amino acid derivatives*

4.1.6.1 L-glutamic acid 5-hydrazide

L-glutamic acid, 5-hydrazide exhibits significant mutagenic activity on E. coli, which qualifies it to function as an antibacterial agent ( Maeda et al., 2021). It plays a role in the bacterial biosynthesis of important antibiotics such as fosfazinomycin and kanamycin ( Wang et al., 2018). This highlights its potential relevance in both medicinal and microbiological contexts.

4.1.6.2 R-noradrenaline

Noreadrenaline, also known as norepinephrine, is a neurotransmitter and hormone that plays a crucial role in the body’s response to stress and blood pressure regulation. It is synthesized from the amino acids tyrosine and phenylalanine ( Dalangin et al., 2020). Norepinephrine primarily functions as a vasoconstrictor, making it an important agent in the treatment of hypotension ( Smith and Maani, 2024).

4.1.6.3 N (alpha)-benzoyl-DL-arginine-p-nitroanilide hydrochloride

N (alpha)-benzoyl-DL-arginine-p-nitroanilide has the capability of enhancing the proteolytic activity of plasmin and trypsin ( Christensen and, Müllertz, 1974; Dulay et al., 2005). The plasmin is involved in the breakdown of the fibrin fibers in blood clots converting them to soluble products ( Famutimi et al., 2024). This compound may be exploited in the treatment of ischemic vascular diseases. Trypsin is a serine protease which facilitates the digestion of proteins and it is involved in progression of colorectal and ovarian cancers ( National Center for Biotechnology Information, 2024k).

4.1.6.4 1-nitroguanidine

Nitroguanidine is likely synthesized from arginine and plays a significant role in the structure of various insecticides and explosives ( National Center for Biotechnology Information, 2024k; Berlinck and Romminger, 2016). The presence of this compound in the structure of honeybees may be attributed to their exposure to insecticides.

*4.1.7 Organometallics and pesticides*

4.1.7.1 Pentaammine (chloroacetato) cobalt (III) diperchlorate

Pentaammine (chloroacetato) cobalt (III) diperchlorate is reacted with 4-Amino-1,2,4-triazole to produce (4-amino-1,2,4- triazole-N1(N2) pentaamminocobalt (III) perchlorate; an explosive material with low toxicity ( Ilyushin et al., 2010). The existence of this organometallic compound in the water extract of the honeybees may be attributed to environmental pollution, presence in insecticide composition or similarity in IR spectra.

4.1.7.2 Potassium diaquabis (malonato) manganite (III)

It is as synthetic organometallic compound with magnetic activity ( Delgado et al., 2006). This compound may found in the water extract of honeybees due to external sources.

4.1.7.3 Alpha-chloralose

Alpha-chloralose is used as pesticides and it is classified as ultra-short acting anesthetic that induces loss of consciousness or as hypnotics and sedatives that induces drowsiness or sleep ( National Center for Biotechnology Information, 2024l).

### 4.2 Effect the water and ethanol extracts on the GTT curves

The normal GTT curve exhibits various patterns, including monophasic (characterized by one peak with subsequent increase and decrease), biphasic (featuring two peaks), triphasic (showing three peaks), and multi-phasic responses. This curve serves as an indicator of the physiological, metabolic, or pathological state of the subjects, whether humans or animals ( de Andrade Mesquita et al., 2018; Vejrazkova et al., 2023).

According to de Andrade et al. (2018), a monophasic GTT curve signals pre-diabetes and pre-metabolic syndrome [104].

The water extract’s impact on the GTT curve is likely attributed to its rich content of phenolic acids and alkaloids, as supported by various studies ( Ali and Siddiqui, 2013; Neelam et al., 2020; Sangshetti et al., 2019; Kumar and Ritika, 2020; Singh et al., 2019; Lin et al., 2016; Kumar et al., 2019). Notably, the presence of five specific compounds in the water extract could help elucidate the observed decrease in glucose levels during the second phase of the water extract GTT curve. The five compounds are 6-methyl-2-benzothiazolamine, 5-(p-aminophenyl)-2-thiazolamine, 2-ethylthio (thiocarbonyl)amino-phenylpropionic acid cyclohexylamine salt, 3-methyl-4-oxo-3,4-dihydro-1-phthalazinecarbohydrazide and 3’-(9-methyl-9H-pyridazino(3,4-b)indol-3-yl) acetanilide ( Table.2 and Table.3).

## 5. Limitations

This study has certain limitations, including the small sample size of experimental rabbits and a somewhat un ideal discussion section (mostly presented as a review) due to the scarcity of published literature on the chemical composition of honeybee bodies

## 6. Conclusions

This study reveals that the aqueous extract of A. mellifera (both drones and workers) contains a broad spectrum of bioactive compounds, including sugar derivatives, phenolic acids, alkaloids, quinones, amino acid derivatives, and short peptides. The administration of this extract induced a notable change in the GTT curve morphology from convex to concave, suggesting anti-hyperglycemic properties. The observed biological activity is likely mediated by specific constituents, particularly dipeptides, phenolic acids, and alkaloids. Consequently, this pilot investigation provides a foundational rationale for expanded research to validate the efficacy of honeybee whole-body extracts in modulating blood glucose levels.

## Data Availability

[Figure Share]. [Glucose Tolerance Test (GTT) Curves of Water and Ethanol Extracts of Whole Body Apis mellifera jemenitica]. [ https://doi.org/10.6084/m9.figshare.29339888] ( Mohammed et al., 2025). This project contains the following underlying data:
• Supplement.1. (Shows the searching the SDBS database). • Supplement.2. (Shows the SDBS search results displaying the 39 compounds detected in the water extract of Apis mellifera jementica). • Supplement.3. (Presents the SDBS search results showing the 12 compounds identified in the ethanol extract of Apis mellifera jementica). • Supplement.4. (Displays the ANOVA test analysis report for the blood glucose level). • Supplement.5. ARRIVE Checklist • Supplement.6. The raw data of the blood glucose level Data is available under the terms of the [CC0].
