# Supplementary material for: Glucose Tolerance Test (GTT) Curves of Water and Ethanol Extracts of Whole Body Apis mellifera jemenitica
Source: F1000Res. 2026 Jan 10;14:872. Originally published 2025 Sep 4. [Version 2] doi: 10.12688/f1000research.166622.2 (PMC12828262; doi:10.12688/f1000research.166622.2)
Supplement: Peer review history

# Reviewer report: Reviewer response for version 2

**DOI:** 10.5256/f1000research.193925.r449132

**Published:** 2026-01-22

**Authors:** semiramis karlıdağ

**Affiliations:** 1 University of Malatya Turgut Ozal, Malatya, Turkey

The authors have satisfactorily addressed all of the comments and suggestions raised during the review process. The revisions have improved the clarity and scientific quality of the manuscript. In its current form, the manuscript is suitable for indexing.

Is the work clearly and accurately presented and does it cite the current literature?

Yes

If applicable, is the statistical analysis and its interpretation appropriate?

Yes

Are all the source data underlying the results available to ensure full reproducibility?

Yes

Is the study design appropriate and is the work technically sound?

Yes

Are the conclusions drawn adequately supported by the results?

Yes

Are sufficient details of methods and analysis provided to allow replication by others?

Partly

Reviewer Expertise:

The article can be published by making some changes I suggested.

I confirm that I have read this submission and believe that I have an appropriate level of expertise to confirm that it is of an acceptable scientific standard.

# Reviewer report: Reviewer response for version 2

**DOI:** 10.5256/f1000research.193925.r449133

**Published:** 2026-01-13

**Authors:** Aida Abd El-Wahed

**Affiliations:** 1 Plant Protection Research Institute, Agricultural Research Centre, Giza, Egypt

The manuscript recommends acceptance in the current form

Is the work clearly and accurately presented and does it cite the current literature?

Partly

If applicable, is the statistical analysis and its interpretation appropriate?

I cannot comment. A qualified statistician is required.

Are all the source data underlying the results available to ensure full reproducibility?

Partly

Is the study design appropriate and is the work technically sound?

No

Are the conclusions drawn adequately supported by the results?

No

Are sufficient details of methods and analysis provided to allow replication by others?

Partly

Reviewer Expertise:

Natural Products Chemistry,  Isolation, purification, synthesis, structure elucidation of bio-active compounds, Bee products, honeybees

I confirm that I have read this submission and believe that I have an appropriate level of expertise to confirm that it is of an acceptable scientific standard.

# Reviewer report: Reviewer response for version 1

**DOI:** 10.5256/f1000research.183639.r422288

**Published:** 2025-12-10

**Authors:** semiramis karlıdağ

**Affiliations:** 1 University of Malatya Turgut Ozal, Malatya, Turkey

In this article, water and ethanol extracts were obtained from the bodies of Apis mellifera jemenitica honeybee drones and workers. The chemical composition of the extracts was investigated. The extracts were also fed to Oryctolagus cuniculus rabbits and their effects on glucose tolerance test (GTT) curves were investigated.

 This is an original and valuable study that will lay the groundwork for the literature. However, as the authors note, the discussion is weak due to the limited material and the scarcity of published literature on the chemical composition of honeybee bodies. I believe it is a valuable study because it paves the way for future studies of this type.

 The method section of the article lacks information on how bee samples were collected and when the analyses were conducted. These missing points are noted in the text.

**Note:** My suggestions and corrections are given above the text.

 It would be more appropriate to use "Chemical composition of honeybee extracts" instead of "Chemical composition of honeybees" in the keywords.

 I made my corrections and suggestions to the article. please find the attached file via following link : https://f1000research-files.f1000.com/linked/762273.my_corrections_and_suggestions_to_the_article.docx

Is the work clearly and accurately presented and does it cite the current literature?

Yes

If applicable, is the statistical analysis and its interpretation appropriate?

Yes

Are all the source data underlying the results available to ensure full reproducibility?

Yes

Is the study design appropriate and is the work technically sound?

Yes

Are the conclusions drawn adequately supported by the results?

Yes

Are sufficient details of methods and analysis provided to allow replication by others?

Partly

Reviewer Expertise:

The article can be published by making some changes I suggested.

I confirm that I have read this submission and believe that I have an appropriate level of expertise to confirm that it is of an acceptable scientific standard, however I have significant reservations, as outlined above.

# Reviewer report: Reviewer response for version 1

**DOI:** 10.5256/f1000research.183639.r425586

**Published:** 2025-11-03

**Authors:** Aida Abd El-Wahed

**Affiliations:** 1 Plant Protection Research Institute, Agricultural Research Centre, Giza, Egypt

**Introduction:**

 - The section beginning with *“The Oral Glucose Tolerance Test (OGTT) consists of several key steps...”* (Eyth et al., 2024) should be omitted; it is unnecessary. The authors should instead discuss the currently used drugs and justify the shift toward exploring natural sources.

**Materials and Methods:**

- The sample size is very small—only four rabbits were used, which were then divided into two groups (n=2). This raises serious concerns about statistical validity.
- The age of the adult workers and drones used should also be specified.
-  The section describing “The study sample consisted of adult workers and drones …… similar to using dry ice for killing honeybees (Mutinelli, 2021).” is overly detailed and unnecessary. The authors should instead provide a clear and detailed description of the extraction method.
-  The doses derived from adult workers and drones are not mentioned. Furthermore, while the authors state there is limited literature on the chemical composition of honeybee bodies, several studies have already addressed this, such as:

1. (Ghosh, et al., 2016) - ref 1
2. (Abedelmaksoud, et al., 2024) - ref 2

**Analytical Methods:**

- Relying solely on IR spectroscopy for compound identification is insufficient. The study should incorporate additional analytical tools such as mass spectrometry (MS) and nuclear magnetic resonance (NMR) to ensure accurate compound identification. Although a list of various compounds is presented, the authors fail to identify which compounds are responsible for the reported biological activity. The authors present list of compounds with diverse of compounds but they didn’t mentionwhich responsible of the activity

**Results and discussions:**

- A more comprehensive discussion of the results is needed. The authors should also include clear recommendations and practical implications of the findings.

**Conclusion**

- Please rewrite the conclusion and include suggestions for future work.
-  The study’s recommendations should be included.

**Figures:**

- All figures should be revised to achieve higher resolution and publication-quality standards.

- The graphic abstract recommended.

Is the work clearly and accurately presented and does it cite the current literature?

Partly

If applicable, is the statistical analysis and its interpretation appropriate?

I cannot comment. A qualified statistician is required.

Are all the source data underlying the results available to ensure full reproducibility?

Partly

Is the study design appropriate and is the work technically sound?

No

Are the conclusions drawn adequately supported by the results?

No

Are sufficient details of methods and analysis provided to allow replication by others?

Partly

Reviewer Expertise:

Natural Products Chemistry,  Isolation, purification, synthesis, structure elucidation of bio-active compounds, Bee products, honeybees

I confirm that I have read this submission and believe that I have an appropriate level of expertise to state that I do not consider it to be of an acceptable scientific standard, for reasons outlined above.

## References

[1] : Nutritional value and chemical composition of larvae, pupae, and adults of worker honey bee, Apis mellifera ligustica as a sustainable food source. *Journal of Asia-Pacific Entomology* .2016;19(2) : 10.1016/j.aspen.2016.03.008 487-495 10.1016/j.aspen.2016.03.008

[2] : Drones and Workers of HoneybeeApis mellifera L. Dried Powder: Chemical Composition, Antioxidant, and Anticancer Assessment. *Food Science & Nutrition* .2024;12(12) : 10.1002/fsn3.4581 10357-10369 10.1002/fsn3.4581 PMC1166682539723047

# Response

**Published:** 2025-12-17

**Authors:** Mohammed Elimam Ahamed Mohammed

**Affiliations:** Chemistry, King Khalid University, Abha, Asir region, Saudi Arabia

**Introduction:**

 Comment: - The section beginning with *“The Oral Glucose Tolerance Test (OGTT) consists of several key steps...”* (Eyth et al., 2024) should be omitted; it is unnecessary. The authors should instead discuss the currently used drugs and justify the shift toward exploring natural sources.

 Response: Thank you very much for your careful review. As the manuscript focuses specifically on the Glucose Tolerance Test (GTT) and related compounds, detailing the GTT procedure is essential for ensuring the accuracy and reliability of the results. Omitting any step would invalidate the findings. I have addressed your point by changing "patient" to "study subject" to better reflect the research context. Thank you again for your valuable feedback.

**Materials and Methods:**

- Comment: The sample size is very small—only four rabbits were used, which were then divided into two groups (n=2). This raises serious concerns about statistical validity.

Response:

1.
  1. We acknowledge that the sample size of experimental animals is limited. This investigation is designed as a pilot study. The decision to use a smaller cohort was guided by ethical considerations, as the potential for serious, life-threatening toxicity from whole-body honeybee extracts in rabbits was previously unknown.
  2. Furthermore, this approach aligns with established ethical frameworks for animal research. Several published articles endorse using minimal animal numbers in preliminary investigations, with some citing 3-5 subjects as an acceptable range for pilot studies, and others noting that certain pilot experiments have been conducted with a single animal. Please see the reference below for further context:

Michael F. W. Festing and Douglas G. Altman. Guidelines for the Design and Statistical Analysis of Experiments Using Laboratory Animals. ILAR Journal. 2002; 43(4): 244- 258.

- Comment: The age of the adult workers and drones used should also be specified.

Response: Done

- Comment: The section describing “The study sample consisted of adult workers and drones …… similar to using dry ice for killing honeybees (Mutinelli, 2021).” is overly detailed and unnecessary. The authors should instead provide a clear and detailed description of the extraction method.

Response: The extraction method was very simple as mentioned and nothing more.

- Comment: The doses derived from adult workers and drones are not mentioned.
- Response: Many thanks. The dose is added to GTT procedure.
- Comment: Furthermore, while the authors state there is limited literature on the chemical composition of honeybee bodies, several studies have already addressed this, such as:
- Response: Thank you very much for your suggestion, however, the published articles are concentrating on the nutritional value alone without other natural products such as phenolic compunds and alkaloids. Beside they did not mention the contaminants such as the pesticides.

1. (Ghosh, et al., 2016) - ref 1, This study is already cited.
2. (Abedelmaksoud, et al., 2024) - ref 2 This study is cited in the introduction and the importance of the study is rephrase accordingly. Thanks the reference is of high importance.

**Analytical Methods:**

- Comment: Relying solely on IR spectroscopy for compound identification is insufficient. The study should incorporate additional analytical tools such as mass spectrometry (MS) and nuclear magnetic resonance (NMR) to ensure accurate compound identification.

Response: While mass spectrometry (MS) and NMR are powerful tools, especially when paired with databases or analysis software, our methodology aligns with standard practices for characterizing complex biological samples. A key aspect of our work was utilizing the new IR spectra database from Japan. Additionally, we are pioneering an approach to use IR spectral peaks much like precursor ions are used to query established metabolomics (e.g., MassBank) and protein databases.

- Comment: Although a list of various compounds is presented, the authors fail to identify which compounds are responsible for the reported biological activity. The authors present list of compounds with diverse of compounds but they didn’t mentionwhich responsible of the activity.

Response: Thanks a lot. In the discussion and conclusion sections we have stated some compounds as possibly responsible for the change of the GTT curve.

**Results and discussions:**

- Comment: A more comprehensive discussion of the results is needed.

Response: This was stated as a limitation because the available literature is limited, with existing research predominantly concentrating on nutritional value.

- Comment: The authors should also include clear recommendations and practical implications of the findings.

Response: Statement is added to the conclusion section to meet your comment.

**Conclusion**

- Comment: Please rewrite the conclusion and include suggestions for future work.
-  The study’s recommendations should be included.

Response: Done.

**Figures:**

- Comment: All figures should be revised to achieve higher resolution and publication-quality standards.

Response: Done according to the journal requirements

- Comment: The graphic abstract recommended.

Response: A graphical abstract is created.
